# Combining acoustic telemetry with archival tagging to investigate the spatial dynamic of the understudied pollack, *Pollachius pollachius*

**DOI:** 10.1111/jfb.15750

**Published:** 2024-04-25

**Authors:** Marine Gonse, Martial Laurans, Justus Magin, Tina Odaka, Jean‐Marc Delouis, Stéphane Martin, François Garren, Coline Lazard, Mickael Drogou, Thomas Stamp, Peter Davies, Alice Hall, Emma Sheehan, Mathieu Woillez

**Affiliations:** ^1^ UMR DECOD (Ecosystem Dynamics and Sustainability) IFREMER, Institut Agro, INRAE Plouzané France; ^2^ UMR LOPS (Laboratory for Ocean Physics and Satellite remote sensing), IFREMER University Brest Plouzané France; ^3^ School of Biological and Marine Sciences, Faculty of Science and Engineering University of Plymouth Plymouth UK

**Keywords:** connectivity, geolocation model, habitat use, hidden Markov model, movement ecology

## Abstract

Combining fish tracking methods is a promising way of leveraging the strengths of each approach while mitigating their individual weaknesses. Acoustic telemetry provides presence information as the fish move within receiver range, eliminating the need for tag recovery. Archival tags, on the other hand, record environmental variables on tag retrieval, enabling continuous path reconstruction of a fish beyond coastal regions. This study capitalizes on the combination of both methods for geolocating pollack, *Pollachius pollachius*, an understudied species of the northeast Atlantic, where declining stocks are raising concern. Essential knowledge of population structure and connectivity between essential habitats is critically lacking and could help inform stock assessment and management. The aims of the study were (1) to evaluate the feasibility of double‐tagging pollack, known for being challenging to tag, and (2) to track seasonal movements across the Channel to gain first insights into pollack spatial ecology. In 2022, an extensive network of acoustic receivers was been deployed in the Channel along the French, English, and Belgian coasts as part of the Fish Intel project. We tagged 83 pollack with acoustic transmitters, among which 48 were double‐tagged with data storage tags. Post‐tagging survival assessment, conducted on a subset of 35 individuals, revealed a successful procedure with a 97% short‐term survival rate. By October 2023, the acoustic telemetry network detected 30 out of 83 pollack at least once, with no large‐scale movements observed across the Channel. Presence in the network fluctuates seasonally, peaking in summer, particularly among immature fish. Integrating acoustic detections with temperature and depth time series in a geolocation model enabled trajectory reconstruction of 10 recaptured pollack, seven of which were detected by the network. This combined tracking approach revealed coastal movements along the coast of Brittany in France, highlighting the ecological significance of the Iroise Sea for pollack throughout the year, particularly in summer. The geolocation model also suggested movements towards the entrance of the western Channel. This study highlights the complementarity of acoustic telemetry and archival tagging in reconstructing fish movements in their natural environment. As data accumulate, these innovative tracking methods promise to continually unveil new insights into the spatial ecology of the understudied pollack, which is essential for the species' management.

## 1 INTRODUCTION

Stock identity is a strong assumption in fish stock assessment, positing that the population within defined boundaries is panmictic, with uniform vital rates (growth, recruitment, and mortality) and no immigration or emigration (Cadrin et al., 2013). However, fish populations often exhibit spatial structure due to the movements of individuals during their life cycle, including larval dispersal, feeding, and spawning migrations (Frisk et al., 2014; Secor, 2015; Waples & Gaggiotti, 2006). These movements disregard the theoretical boundaries imposed by stock assessment (Reiss et al., 2009), and may overlap in space and time with human activities at sea, particularly fisheries (Ciannelli et al., 2013). Ignoring the heterogeneity in spatial population dynamics in the assessment‐management process could lead to inappropriate exploitation because it might skew population vital rates and render management measures unsuitable (Goethel et al., 2016). In cases of reproductive isolation within a population and heterogeneous exploitation rates, there is a potential for overexploitation of less productive subunits and underexploitation of more productive ones, hindering the sustainable exploitation of marine resources (Cadrin & Secor, 2009; Fu & Fanning, 2004; Goethel & Berger, 2017; Ying et al., 2011). While the necessity to incorporate spatial information in stock assessment (e.g., spatially explicit stock assessment) and management (e.g., marine protected areas, spatial planning, real time closures of fisheries) is widely recognized (Cooke et al., 2016; Hays et al., 2019), its practical implementation has been limited by the mathematical complexity of associated models and the scarcity of movement data for numerous fish populations (Punt, 2019).

Observing and tracking marine animals in their environment is challenging, especially fish as they continuously swim underwater. However, various approaches have been developed to monitor fish movements. We are currently in the midst of the biologging decade (2018–2028), characterized by rapid advancements in biologging devices that enable the study of marine animals' trajectories and their surrounding environment (Lowerre‐Barbieri et al., 2019). Among the wide range of electronic tags used for fish tracking, archival tagging and acoustic telemetry are two commonly employed technologies.

Archival tags are extensively used for inferring habitat use, migration pathways, connectivity patterns, and population structure by reconstructing fish trajectories based on remotely measured environmental variables (e.g., temperature, depth, light intensity, tide). The principle is to estimate probabilities of fish positions by comparing tag records with field observations or model outputs of the same variables within a hidden Markov model (HMM) framework (Liu et al., 2017; Pedersen et al., 2008; Woillez et al., 2016). The HMM allows for the explicit modeling of movement and measurement processes, as well as their respective errors. Archival tags have the advantage of providing continuous fish tracking as environmental data are recorded at predefined time intervals (e.g., a few seconds to minutes). However, archival tags must be recovered to access data (e.g., data storage tags) or transmit their data via satellite (e.g., pop‐up satellite archival tags), potentially hindering the ability to collect a sufficient amount of tracking data to extract representative movement patterns within a fish population. In addition, geolocation model performance may vary according to reference field heterogeneity and grid resolution, for instance in coastal areas where strong temperature gradients occur, leading to potential inaccuracies and errors around fish location estimates (Nielsen et al., 2019). The use of complementary techniques, such as acoustic telemetry and archival tagging, can partly overcome the limitations of geolocation modeling approaches (Goossens et al., 2023).

Acoustic telemetry is a tracking method that relies on networks of acoustic receivers that provides insights into fish movement patterns, habitat use, and residency (Hussey et al., 2015). An acoustic transmitter, either implanted or attached to the fish, emits a unique identifier at regular intervals. This enables receivers to record time‐stamped detections as the fish swims within their detection range. Unlike archival tags, the receivers store records, allowing for the collection of fish presence data without requiring recapture (Cooke et al., 2012). In addition, acoustic telemetry can provide information on the fish's exact location in dense arrays of receivers where triangulation is possible (e.g., Baktoft et al., 2017; Whoriskey et al., 2022). However, acoustic telemetry does not allow exhaustive monitoring of fish movements at large spatial scales as building an extensive and dense array of receivers would be costly and unrealistic. Most of the time this tracking technology is limited to coastal areas, where deploying receivers is convenient.

Double‐tagging involves attaching two tags to a fish. By combining two tracking methods, one can capitalize on the advantages of each specific method while mitigating their individual weaknesses (Begg & Waldman, 1999). Among the possible tag combinations, the joint use of archival and acoustic tags enables the comparison of acoustic detections and estimated locations derived from geolocation models, ultimately refining the reconstruction of fish trajectories (Gatti et al., 2021; Goossens et al., 2023). Combining archival and acoustic tags is particularly relevant for fish populations that frequent both coastal and offshore habitats as networks of acoustic receivers in coastal regions can capture coastal movements (e.g., Cooke et al., 2011; Taylor et al., 2017; Abecasis et al., 2018; Reubens et al., 2019) while archival tags can help reconstruct fish movement beyond acoustic telemetry networks (e.g., Woillez et al., 2016).

Pollack, *Pollachius pollachius* (Linnaeus, 1758), is a bentho‐demersal species from the Gadidae family distributed in the northeast Atlantic from Portugal to Norway. Juveniles are coastal during their first 2 years, while adults are found offshore at depths between 40 and 100 m (Pawson, 1995). Reproduction occurs between February and May from the Iberian coast to the Celtic Sea and extends up to June along the Norwegian coast (Moreau, 1964). Mature individuals (*L*_maturity_ = 43.71 cm; Alemany, 2017) aggregate during this period (Suquet, 2001). Pollack has high economic and cultural value in Spain, France, the UK, and Norway, where it is exploited by both professional and recreational fisheries. The International Council for the Exploration of the Sea (ICES) has defined three units for the purpose of management and advice on fishing opportunities for pollack: the Bay of Biscay and Atlantic Iberian waters (subarea 8 and division 9.a), the Celtic Sea and the English Channel (subareas 6 and 7), and the North Sea, Skagerrak and Kattegat (subarea 4 and division 3.a). Currently, the Celtic Sea and English Channel stock is considered data‐limited (ICES category 3 stock) given the lack of information regarding the identity of the stock and the lack of fishery‐independent abundance indices (ICES, 2023b). Landings in the Bay of Biscay and Atlantic Iberian waters decreased over the 1990s and have stabilized at around 2000 tonnes/year in recent years (ICES, 2023a). A similar trend has been observed in the North Sea, Skagerrak and Kattegat stock (ICES, 2021). In the English Channel, however, landings have dramatically dropped since the 1990s and the decline has accelerated since 2014. According to the latest report of the ICES advisory committee for pollack in the Celtic Seas, a zero catch recommendation has been made for 2024 (ICES, 2023b). This urges the need for a better understanding of pollack spatial dynamics in the northeast Atlantic.

Moreau (1964) evidenced differences in morphometric characteristics of pollack from the Iberian coast compared to pollack from the Bay of Biscay and English Channel. Decades later, Charrier et al. (2006) investigated the spatial structure of pollack populations in the northeast Atlantic using micro‐satellites. The authors evidenced a very low but significant genetic differentiation between pollack from the southern Bay of Biscay, the western Channel, and the northern North Sea. However, they suggested that further research is required to confirm this finding given the small sample sizes and the limited number of loci used. The stable trends of landings in the Celtic Sea and Bay of Biscay over recent years, as opposed to the strong decline observed in the English Channel, might suggest different dynamics between the English Channel and adjacent units (ICES, 2017), in line with the results from Charrier et al. (2006). More recently, Alemany (2017) developed a Bayesian hierarchical model to estimate growth and maturity parameters in the English Channel, the Bay of Biscay, and Iberian waters. Alemany found relatively similar parameters between the English Channel and the Bay of Biscay units. However, he also stated that inferring the stock identity using solely the life‐history traits might be misleading. Consequently, the stock definition of pollack in the northeast Atlantic remains unresolved and information on the movements of pollack is required to assess the relevance of the stock boundaries, especially on both side of the 48° parallel separating the English Channel from the Bay of Biscay (Foucher, 2017; ICES, 2017).

Pollack is a challenging species to tag given its propensity to undergo barautrauma due to rapid depressurization after being caught in deep waters, which can reduce post‐release survival. The first successful tagging experiments on pollack took place in 1979 in Norway using external tags (Jakobsen, 1985). More recently, acoustic telemetry studies have been conducted on pollack in coastal Skagerrak (Freitas et al., 2021) and in Spanish waters (Mucientes et al., 2021). These two studies have demonstrated the viability of tagging pollack. However, both studies covered relatively small coastal areas, and larger‐scale tagging experiments are required to analyze the spatial population dynamics of pollack, particularly in the Channel, where there is a concerning decline in landings. The present study investigated pollack movements across the Channel by coupling archival tagging and acoustic telemetry. This study aimed to (1) assess the feasibility of double‐tagging for pollack and (2) track seasonal movements of pollack by analyzing the timing of acoustic detections and by running a geolocation model fed with both archival and acoustic telemetry data. Note that our goal was not to estimate the impacts of double‐tagging on pollack in terms of growth or behavioral changes, but rather to assess its feasibility by using acoustic detections and recapture events as proxies of fish survival in the short and long terms.

## 2 MATERIALS AND METHODS

### 2.1 Tagging protocol

The tagging protocols varied slightly between the French and English tagging locations due to the specific characteristics of both the sites and fishing techniques.

In France, the tagging procedure involved a fishing vessel and a support vessel for surgery and stalling. On the fishing vessel, professional rod and line fishers utilized their ecological knowledge to catch pollack at locations with depths not exceeding 15 m, aiming to maximize survival and minimize barotrauma. In case of swim bladder inflation due to rapid depressurization, excess gas was released via insertion of a hypodermic needle. Fish were carefully chosen based on specific criteria, including total length >38 cm, a tag‐to‐body‐mass ratio of <2%, and apparently good condition. The maximum total length of selected fish was 62 cm and the mean total length was 47 cm. These fish were then transferred to the tagging vessel and placed in a 600‐L tank supplied with flowing seawater for several hours before undergoing further treatments.

**FIGURE 1:**
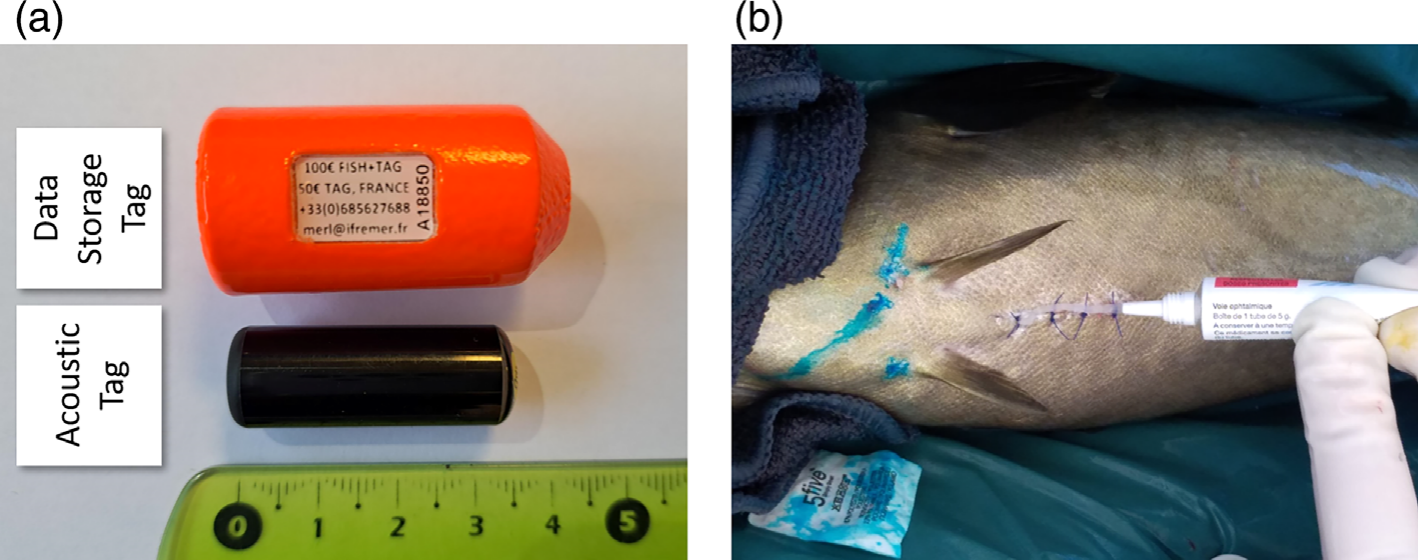
(a) The data storage tag (DST) and acoustic tag used to track pollack. (b) Application of an antibiotic treatment on a sutured wound after tag insertion inside the peritoneal cavity of a pollack. Blue spots correspond to the intradermal injection of alcian blue between the pectoral fins. Credit photo: IFREMER.

Anesthesia was administered using Iso‐Eugenol (at a concentration of 80 ppm) for an average duration of 3–4 min. Prior to the procedure, the total length and weight of the fish were measured. The fish were then placed on an operating table where water and Iso‐Eugenol (at a sedation dose of 15 ppm) flowed through their gills, and a damp towel was positioned over their eyes to reduce stress. A caudal fin clip measuring 1 cm^2^ was sampled for further genetic investigations. The incision site, located beyond the pelvic fins, was disinfected with Betadine. Following the incision, one (acoustic tag) or two (acoustic tag and data storage tag, for pollack exceeding 950 g) tags were implanted in the peritoneal cavity (Figure 1a). The wound was then sutured, and an antibiotic treatment (Tobrex) was applied to the wound (Figure 1b). Subsequently, an analgesic (Lurocaine) was administered via intramuscular injection (0.05 mL/kg). To facilitate external identification of tagged fish, they were tattooed using an intradermal injection of alcian blue between the two pectoral fins with a Dermojet device (Figure 1b). Following surgery, fish were transferred to an aerated recovery tank. They were monitored visually until they resumed normal swimming behavior, and they were released carefully 24 h later. Ethical guidelines were strictly observed and all tagging procedures were conducted under the project license APAFIS#32389–201070816107734*v5*, which was authorized by the French Ministry of Higher Education, Research and Innovation.

In the UK, the tagging protocol slightly differed. Only one vessel was responsible for both fishing and tagging, and fish were captured at 25 m depth on average. After capture, they were immediately placed in an anesthetic. Anesthesia was administered via immersion within tricaine methanesulfonate (at a concentration of 80 mg/L) for an average duration of 3 min. Prior to the procedure, the fork length was measured and excess gas released from the swim bladder via insertion of a hypodermic needle. The fish were then placed on a V‐shaped cradle and an incision made adjacent to the mid line close to the pectoral fins. An acoustic transmitter was inserted within the peritoneal cavity. The wound was sealed using an interrupted suture, closed with a surgeon's knot. Lidocaine was topically applied to the surgical site to provide post‐operative analgesia. Once the tag was implanted, the fish were monitored to ensure that they recovered from the anesthetic, and then quickly returned to the water. The fish were kept on board the boat for no longer than 10 min in total. All tagging procedures in the UK were conducted under Home Office License P81730EA5.

### 2.2 Tags and receivers

#### 2.2.1 Archival tags

Double‐tagging using an acoustic tag and a data storage tag (DST) exclusively occurred in France. The DSTs used were Cefas G5 long‐life 2 Mb models, with a diameter of 21 mm, a length of 36.5 mm, and a weight of 2.5 g in saltwater (excluding the float) (Figure 1a). These tags were equipped with temperature and pressure sensors and had a battery lifetime of 2 years. They were calibrated to record pressure within a range of 0–24 bars, allowing for a maximum depth record of 230 m. Temperature and depth data were recorded every 90 s.

To improve the chances of recovering deployed DSTs, floating tags were used. These tags had the ability to drift ashore in the event that the tagged fish died at sea, regardless of the cause of death. Although we could not guarantee that internal floating DSTs had no detrimental effect on fish movements and survival, we assumed that any potential effects were likely limited compared to the drag imposed by external floating tags, such as pop‐up satellite tags (Hedger et al., 2017). Despite the positive buoyancy of our DST, it is unlikely that this significantly affected the swimming ability of the fish, as the buoyancy was relatively small in comparison to the fish's body mass. Indeed, we strictly observed the 2% tag‐to‐body‐mass ratio, ensuring that no fish weighing under 950 g were equipped with DSTs.

In France, efforts were made to promote the experiments and encourage the return of tagged fish and DSTs. This included advertising through various media channels such as newspapers and radio, as well as using posters and mailings to reach out to fishers and stakeholders. A reward of 100€ was offered for each tagged fish returned to the laboratory, with a reward of 50€ for the return of a DST alone.

#### 2.2.2 Acoustic tags

In France, the acoustic tags used were Thelma Biotel MP13‐ID with a frequency of 69 kHz, a weight of 7.1 g in water, a diameter of 13 mm, and a length of 33 mm (Figure 1a). These tags were configured to emit signals following the Open Protocol (OPi), as recommended by the European Tracking Network (https://europeantrackingnetwork.org/en/open-protocol; Reubens et al., 2021).

The same Thelma Biotel acoustic tags were employed in the UK but these tags were configured to emit signals based on an older protocol known as R64K. This choice was made to ensure compatibility with existing tag protocols and equipment in UK waters.

Both types of tags emitted a unique identification code with a random delay of 3 min on average. They were designed to function for a duration of up to 5 years.

#### 2.2.3 Acoustic receivers

In France, the acoustic receivers used were Thelma Biotel TBR 800 RELEASE operating at 69 kHz. These receivers were equipped with a buoy and anchored with a weight ranging from 75 to 100 kg near the sea bottom, with the receivers oriented upwards. The transmitter tag range was estimated to be approximately 400 m. The Thelma Biotel acoustic receivers were configured to monitor six different communication protocols, including the Open Protocol (OPi, OPs, R64K, R01M, S256 and S64K).

In the UK, the acoustic receivers utilized were Innovasea VR2AR operating at 69 kHz. All receivers were anchored to the seabed with 60 kg ballast, orientated upwards. Transmitter tag range was estimated to be approximately 300 m. Innovasea receivers were configured to detect MAP114 compatible code maps, for example R64K.

Pollack equipped with acoustic tags in UK waters (parameterized in R64K) could be detected by both the Innovasea and Thelma Biotel receivers. Conversely, pollack fitted with acoustic tags in French waters (parameterized in OPi) could only be detected by acoustic receivers of the same manufacturer in French waters. This compatibility issue limited our ability to track pollack as they moved across the Channel from French to English waters.

### 2.3 Headcount of tagged fish and acoustic stations

**FIGURE 2:**
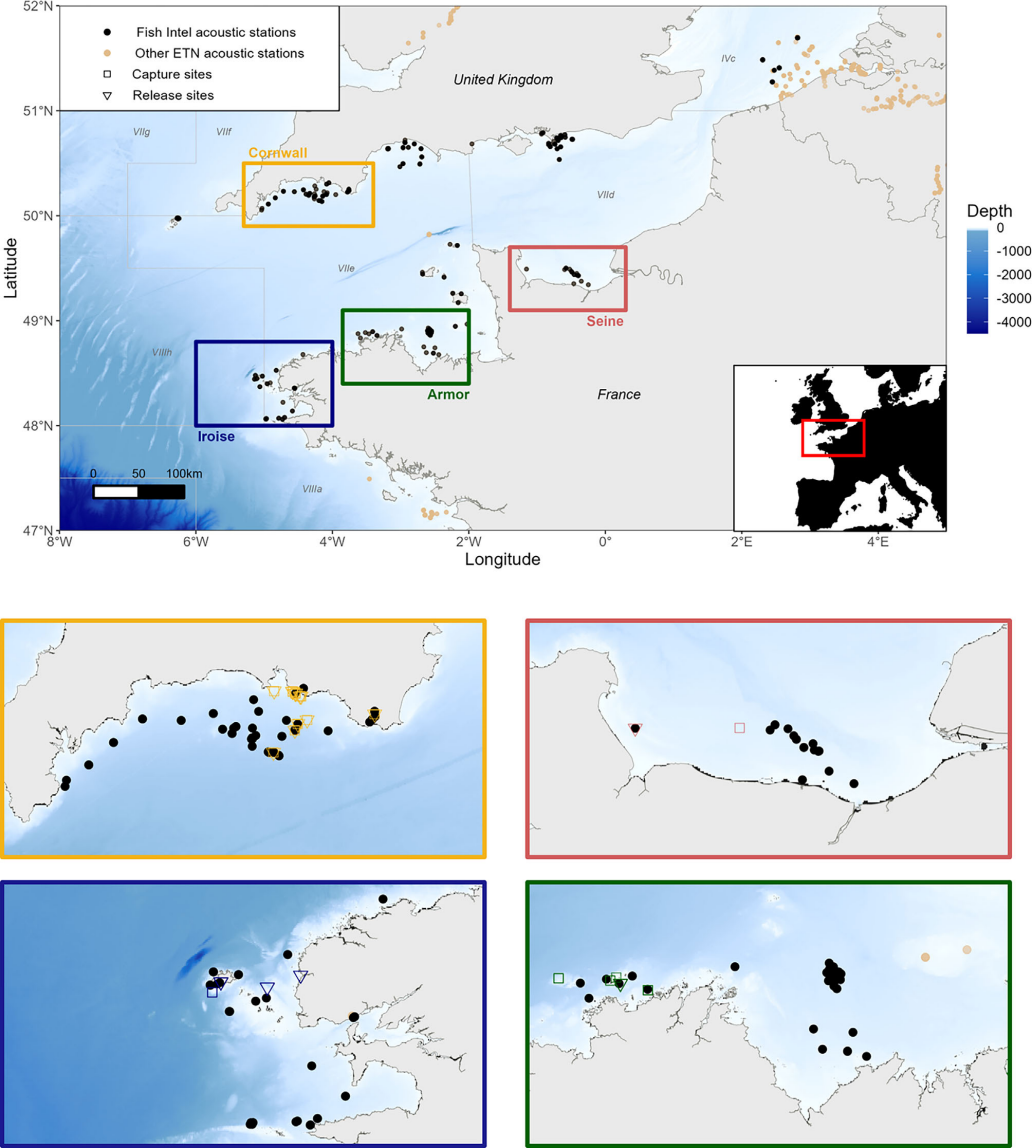
Map of the pilot sites where pollack were captured, tagged, and released during the Fish Intel project. Dots indicate acoustic stations where receivers were deployed during the Fish Intel project or the acoustic telemetry project hosted by the European Tracking Network (ETN).

Four pilot sites were defined in the Channel where pollack were likely to be found: Iroise Sea (referred to as Iroise), Côtes d'Armor (referred to as Armor), and Bay of Seine (referred to as Seine) in France, and Cornwall and South Devon (referred to as Cornwall) in the UK (Figure 2). The locations of acoustic stations were determined in collaboration with professional fishers to maximize the likelihood of detecting fish.

In 2022, a total of 83 pollack were tagged, with 70 and 13 individuals tagged in the French and UK waters, respectively (Table 1).

**TABLE 1:** Numbers of pollack tagged, fish total length, and acoustic stations deployed and serviced as of October 2023 in each pilot site.

Pilot sites	Fish equipped with acoustic tags (+ DST)	Mean and range of fish total length (mm)	Acoustic stations deployed	Acoustic stations serviced
Iroise	55 (37)	508 [386–622]	29	23
Armor	14 (11)	486 [408–560]	33	27
Seine	1 (0)	430	18	14
Cornwall	13 (0)	420 [350–620]^a^	89	72
	**83 (48)**	**430 [350–622]**	**169**	**136**

^a^ Fork length.

On the French side, there could be variations in the location and date of release compared to the location and date of capture due to the tagging protocol, which involved two vessels operating over several days. In the Iroise Sea, 55 individuals were captured, tagged, and released between June 12 and 17, 2022, at three different locations. In the Côtes d'Armor, 14 individuals were captured, tagged, and released between May 17 and 22, 2022, at a single location. Additionally, one individual was captured, tagged, and released in the Bay of Seine on May 27, 2022 (Figure 2).

On the English side, the capture, tagging, and release events took place simultaneously, as only one vessel was engaged in the entire procedure. Individuals tagged in Cornwall and south Devon off Plymouth were captured and released on various dates in 2022. Specifically, there were releases on January 25, 2022 (one individual), January 26, 2022 (one individual), February 11, 2022 (eight individuals), September 12, 2022 (one individual), and September 16 2022 (two individuals) (Figure 2).

Three out of 83 tagged individuals were considered presumably dead since their tags were detected by acoustic receivers continuously over the study period in 2022 (one individual tagged in the Iroise Sea and two individuals tagged in south Devon).

### 2.4 Acoustic data filtering

Acoustic detections were stored on the ETN database (Reubens et al., 2019). Essentially, acoustic detections consist in a tag ID, a timestamp, a receiver ID, a station name, and geographical coordinates. To extract these acoustic detections along with the receivers, tags, and animal's metadata from the ETN database, we used the ETN RStudio server and the etn R package (Desmet et al., 2022).

False detections resulting from signal collision or background noise can lead to erroneous indications of animal presence. We developed a filtering procedure to remove such false detections by adapting a method described by Hoenner et al. (2018). We established a set of quality control (QC) criteria. Each detection was scored based on criteria of isolation, velocity and distance between consecutive detections, distance between detections and release location, release date, and signal to noise ratio. See Appendix A in the Supporting Information for the details of the filtering procedure.

### 2.5 Post‐tagging survival

**FIGURE 3:**
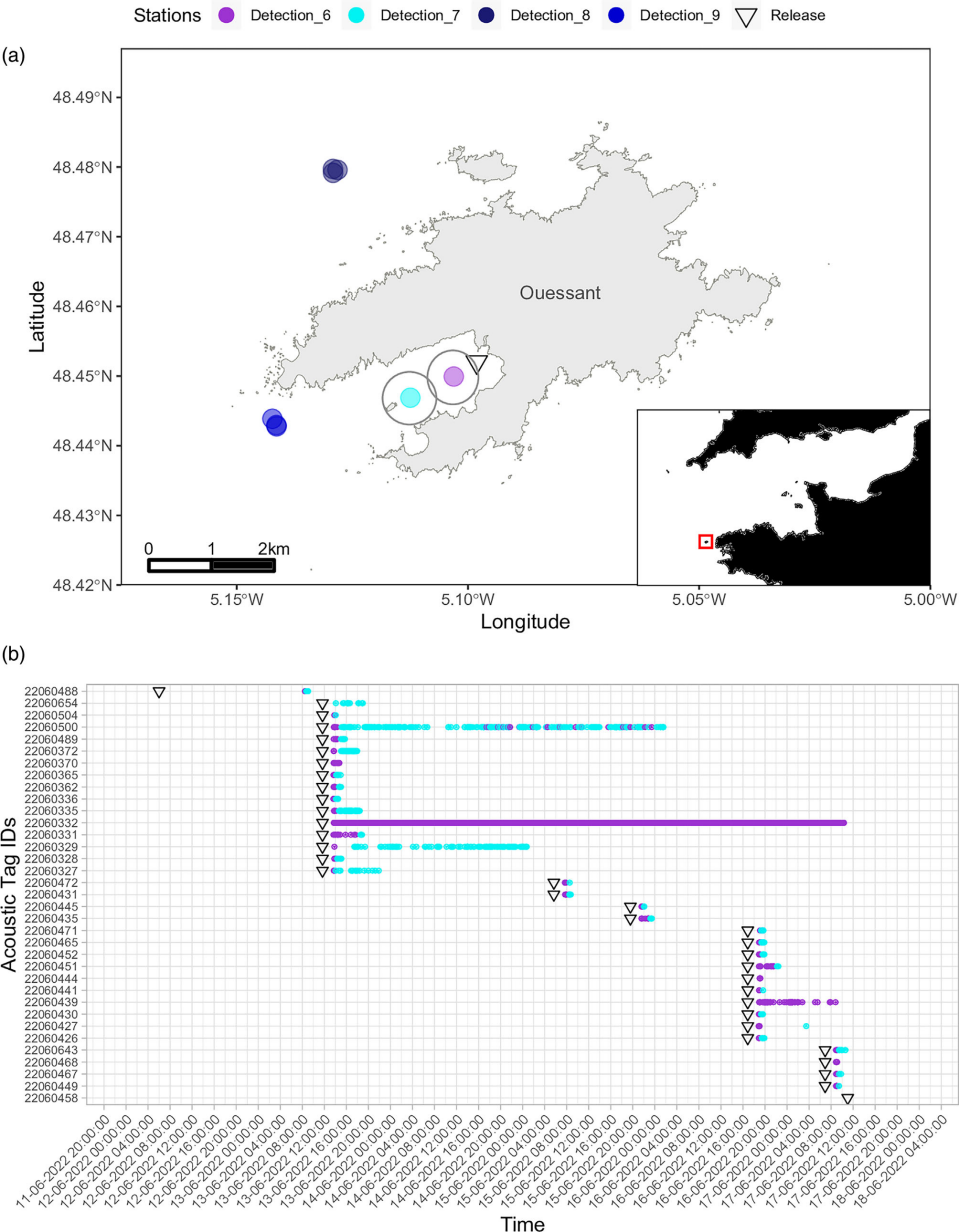
(a) Map of temporary and permanent receivers deployed nearby Ouessant Island. Detection_6 and Detection_7 were temporary receivers placed in the vicinity of the release location in the Bay of Lampaul between June 12–17, 2022. The detection ranges of the two temporary receivers are indicated by the gray circles. (b) History of release date times and acoustic detections of pollack tagged and released in the Bay of Lampaul, Ouessant Island, between June 12 and 17, 2022.

Using the acoustic detections and recapture events as proxies of survival, we conducted two distinct post‐tagging survival analyses. The assessment of long‐term survival involved calculating the percentage of fish that were detected and/or recaptured after spending at least a week at sea. In parallel, for the purpose of evaluating the short‐term post‐tagging survival of pollack, we deployed temporary receivers at two distinct locations near one of the release sites, within the Bay of Lampaul off Ouessant Island in the Iroise Sea, France. These receivers were designated as Detection_6 and Detection_7 (Figure 3a). A total of 35 out of the 55 pollack tagged in the Iroise Sea were released into the bay, and we closely monitored their movements as they made their way out of the bay. The two receivers were strategically positioned 700 m apart, ensuring that their respective detection ranges did not significantly overlap. This setup enabled us to sequentially track the fish as they progressed towards the exit of the bay.

### 2.6 Residency periods

Gaps between acoustic detections obtained from fixed receivers not only enable the tracking of fish movements between acoustic stations but also provide valuable insights into the residency behavior of fish in specific areas (Williamson et al., 2021). To assess this behavior, we computed the duration between consecutive detections at each acoustic station for each individual fish. Subsequently, we defined residency periods based on these time intervals. If two consecutive detections occurred within less than a predefined residency period (e.g., 24 h), they were considered part of the same residency period. Otherwise, they were regarded as independent residency periods. We then calculated the number of residency periods for each individual fish and explored the relationship between this number and the fish's total length, which served as a proxy for the fish's developmental stage. We conducted a sensitivity analysis of the threshold for residency periods (i.e., from 1 to 24 h) as presented in Appendix B in the Supporting Information.

Note that this analysis excluded data from the two temporary receivers, Detection_6 and Detection_7, which were deployed during the tagging experiments. This exclusion was necessary because residency periods inferred from the acoustic detections at these locations would be more influenced by the tagging event than the natural behavior of pollack.

### 2.7 Geolocation model

Individual trajectories were reconstructed using a hidden Markov model (HMM) framework for every recovered DST. The HMM allows inference of fish hourly positions (hidden states) by combining a movement model (typically a Brownian random walk) with an observation model that relates sensor records (here the temperature and depth) with geophysical reference fields (Pedersen et al., 2008). A geolocation model based on temperature and depth data was previously developed for tracking European seabass by Woillez et al. (2016). Here, we adapted this model to integrate acoustic detections in addition to temperature and depth records following the method used by Goossens et al. (2023).

The geophysical reference fields consisted in the Atlantic—European North West Shelf—Ocean Physics Analysis, a hydrodynamic model based on a eddy‐resolving Nucleus for European Modelling of the Ocean model application at a horizontal curvilinear grid of 1/36 degrees of horizontal resolution and 50 vertical levels (CMEMS, 2023). The data we used from this hydrodynamic models are the hourly mean sea surface height above geoid, hourly mean sea water temperature, and the bathymetry derived from GEBCO 08 (30 arc‐second resolution). The domain was limited to 8°W–1°W in longitude and 47° N–51° N in latitude to reduce computing resources. The geophysical reference field's resolutions in this region corresponds to approximately 2 × 2 km. The HMM model was optimized using the Pangeo ecosystem to ease the intensive data handling and computing (pangeo‐fish open software; Magin et al, inprep).

A detection likelihood was used to constrain the location predictions from sensor data (depth and temperature) with presence absence observations from acoustic detections. At a hourly time step, if a fish was detected by a receiver, the likelihood was set to 1 for the corresponding grid cell and 0 for the other cells. If the fish was not detected by any of the receivers, the likelihood was set to 0 in the corresponding cells and a non‐null value for the rest of the cells.

As proposed in Goossens et al. (2023), we assessed model performance by calculating the track sensitivity as the distance between the positions inferred from the geolocation model including acoustic detections and the positions inferred from the geolocation model excluding acoustic detections.

## 3 RESULTS

### 3.1 Post‐tagging survival

#### 3.1.1 Short‐term survival

**FIGURE 4:**
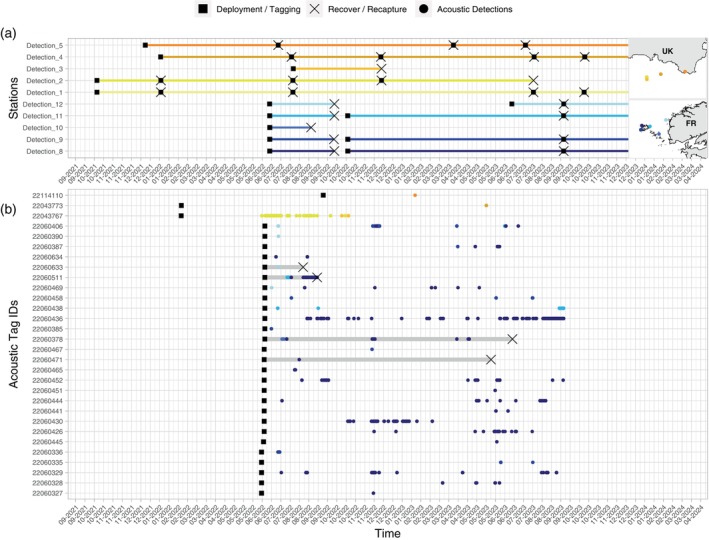
History of acoustic receivers and tagged pollack between May 2022 and September 2023. (a) Deployment and recovery of acoustic receivers. (b) Tagging, detections, and recapture of pollack. Gray horizontal lines highlight the history of fish recaptured. The maps indicate the positions of the stations in the Cornwall and Iroise pilot sites.

In the Bay of Lampaul, 34 out of 35 released pollack were detected by the temporary stations Detection_6 and Detection_7 (Figure 3). Fish 22060458 was not detected inside the Bay but was detected a few weeks after at another station near Ouessant Island (Figure 4). Fish 22060332 presumably died after tagging, as indicated by the continuous detections at station Detection_6 (Figure 3). Except for fish 22060654, which was only detected at stations Detection_6, individuals detected inside the Bay of Lampaul were sequentially identified at stations Detection_6 and then at Detection_7, i.e., approximately 1.5 km away from the release site. Considering the low currents inside the Bay of Lampaul and the sensitivity of pollack, which would likely die immediately after release in case of trauma following tagging, these results suggest that 34 pollack were able to recover and escape the Bay of Lampaul. This indicates a short‐term post‐tagging survival rate of 97% (Figure 3). Out of the 35 pollack released inside the Bay of Lampaul, 16 were later detected outside the Bay and 3 were recaptured after a week at sea, indicating that at least 54% of those fish survived in the long‐term.

Among the 35 pollack tagged and released in the Bay of Lampaul, all the double‐tagged fish (*n* = 21) were detected. The individual that presumably died (fish 22060332), was tagged with a single tag (acoustic tag), therefore the short‐term survival rate was very similar between the fish tagged with one (acoustic) or two (acoustic and DST) tags.

#### 3.1.2 Long‐term survival

Among the 70 pollack tagged in France, 27 were detected by the acoustic telemetry network (excluding the temporary stations Detection_6 and Detection_7) after a week or more. In addition, five double‐tagged individuals tagged in France were recaptured by professional or recreational fishermen without being detected by the acoustic telemetry network. As a result, 32 (46%) individuals tagged in France survived in the long‐term. Among the 13 pollack tagged in the UK, three were detected by the acoustic telemetry network (23%). In summary, a total of 35 pollack tagged in France and the UK were considered alive after at least a week at sea, representing a 42% long‐term survival rate.

Among the 83 tagged pollack, 16 out of the 47 double‐tagged pollack were detected (34%), and 14 out of the 36 pollack tagged solely with an acoustic tag were detected (39%). Consequently, the detection rates of pollack tagged with one (acoustic) or two (acoustic and DST) tags were remarkably similar, suggesting that the survival rate was unaffected by double‐tagging.

### 3.2 Movements inferred from acoustic detections

#### 3.2.1 Overall movement patterns

Hereafter, acoustic detections obtained from the two temporary stations, Detection_6 and Detection_7, have been excluded from the movement analysis since this location information were redundant with the release locations. As of October 2023, 30 out of the 83 tagged individuals have been detected at least once within the acoustic telemetry network (Table 2, Appendix C in the Supporting Information). Six out of 13 immature tagged pollack were detected, while 21 out of 57 mature tagged pollack were detected. Most of these detections were recorded in the Iroise Sea, with fewer occurrences in Cornwall (Figure 4). As expected, no movement has been observed between the French and English pilot sites. Additionally, no fish tagged in the Côtes d'Armor or the Bay of Seine was detected within the network. Nine individuals have been recaptured in the Iroise Sea, among which eight were initially tagged in the same area, indicating residency behavior within the Iroise Sea. In addition, among the nine individuals recaptured in the Iroise Sea, one was tagged in the Côtes d'Armor (fish A19230/22060608), suggesting westward movement along the coast of Brittany during the summer of 2022. Finally, one individual tagged in the Côtes d'Armor had its tag recovered on a beach near the tagging site a year later (fish A19226/22060612). Among the 10 recaptured fish, five were also detected in the Iroise Sea, excluding the detections at the temporary stations Detection_6 and Detection_7 (Figure 4).

**TABLE 2:** Number of receivers detecting pollack and number of pollack detected and recaptured (note that all the recaptured fish were double‐tagged)

Pilot sites	Receivers with detections	Fish detected at least once	Fish recaptured
Iroise	5	27	9
Armor	0	0	1
Seine	0	0	0
Cornwall	5	3	0
	10	30	10

In the UK, the listening effort of the acoustic network was relatively stable, ranging from 2204 to 2800 cumulative days per month across the deployed receivers in Cornwall.

**FIGURE 5:**
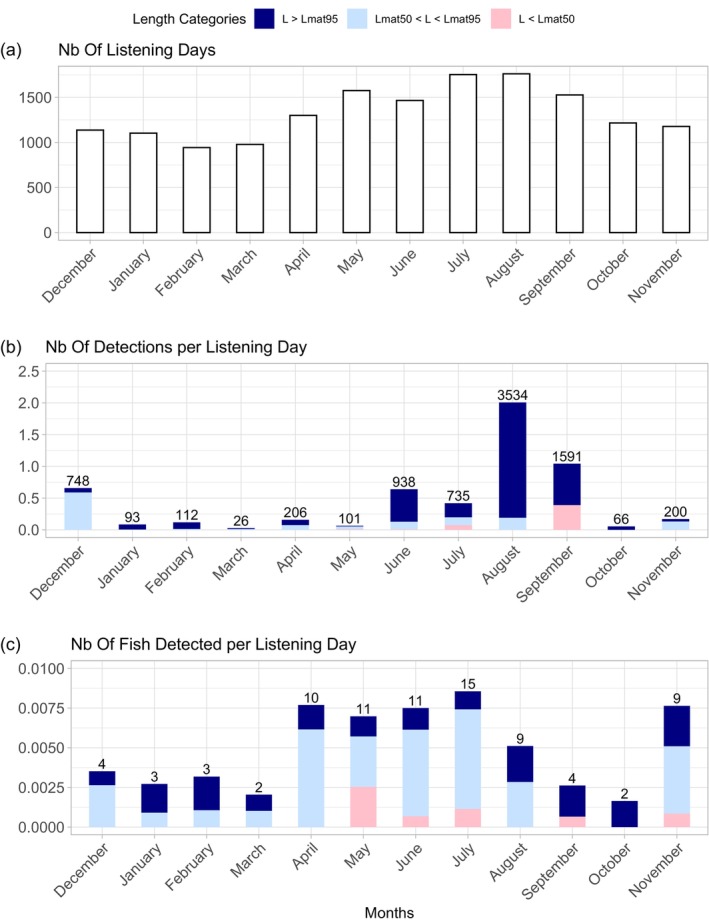
(a) Number of listening days per month across the telemetry network in Iroise, Armor and Seine. (b) Number of detections per listening day calculated per month and fish length categories. The numbers above the bars indicate the total number of detections. (c) Number of individuals detected per listening day calculated per month and fish length categories. The numbers above the bars indicate the total number of individuals detected. The length at maturity *Lmat*_50_ = 43.7 cm and *Lmat*_95_ = 55.3 cm were retrieved from Alemany (2017).

In France, the listening effort of the acoustic network varied, ranging from 943 to 1761 cumulative days per month across all the deployed receivers in the Iroise Sea, Côtes d'Armor, and Bay of Seine (Figure 5a). Since the deployment of the receivers began in May 2022 and the final service was conducted in September 2023, the number of listening days was higher during the spring and summer months (i.e., two summers were monitored from May 2022 to September 2023). This seasonality in listening effort correlated with the seasonality of the number of detections (*R* ^2^ = 0.3294, *F* = 6.404, *p* = 0.0298), with an increase in detections per day between June and September, as well as in December (Figure 5b).

Regarding the number of individuals detected per day, despite the relatively low number of detections in April and May, a substantial number of fish were detected (Figure 5c). This suggests that individuals were present but potentially too mobile to be consistently detected during this period. In contrast, August exhibited an inverse pattern, with the highest number of daily detections observed alongside a moderate number of fish detected, suggesting high residency of few individuals in the vicinity of the receivers.

Immature individuals (total length under *Lmat*_50_ = )^1^ were only detected from May to November, except in August and October (Figure 5b,c). This suggests that immature and mature fish may exhibit different residency patterns in the vicinity of acoustic receivers throughout the year.

#### 3.2.2 Residency periods

Fish 22060436 and 22060438 were presumably dead at the end of the detection time series (Figure 4), considering that the average duration between successive detections was 3 min, i.e., the average ping frequency of the acoustic transmitters. Consequently, the detections corresponding to their last residency periods were removed from the following residency analysis.

**FIGURE 6:**
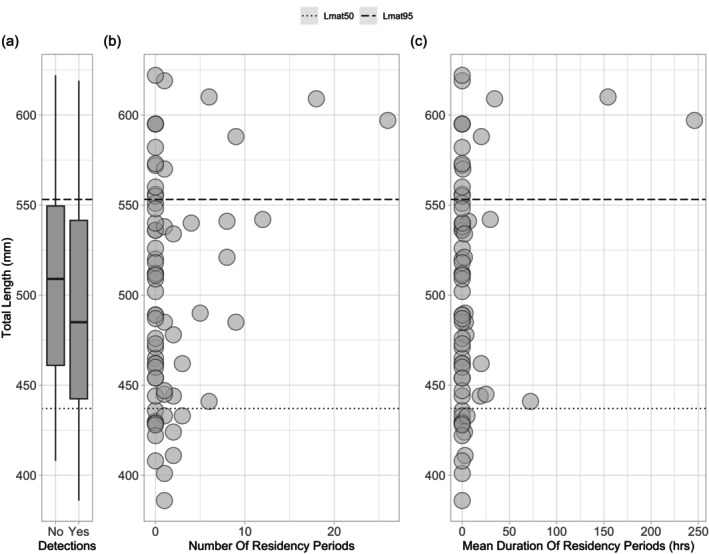
(a) Total length of fish detected (*N* = 27) and not detected (*N* = 43) in the Iroise Sea, Côtes d'Armor, and Bay of Seine. (b) Relation between total length and number of residency periods inferred from acoustic detections. The threshold used to compute the residency periods was fixed to 24 h. (c) Relation between total length and mean duration of residency periods. The length at maturity *Lmat*_50_ = 43.7 cm and *Lmat*_95_ = 55.3 cm were retrieved from Alemany (2017).

We found no significant difference of total fish length between individuals detected and not detected in the Iroise Sea, Côtes d'Armor, and Bay of Seine (Figure 6a). However, the number and duration of residency periods seemed to increase with fish total length (Figure 6b,c). The same pattern was observed whatever the duration threshold used to define the residency periods (Appendix B in the Supporting Information). The mean number of residency periods among the detected individuals was five, with a mean duration of 24.2 h. Given that the receivers were positioned in coastal areas (<10 km from the coast, between 20 and 60 m depth) and their detection range covered a relatively limited part of the coastline, this outcome suggests that the receivers were positioned in coastal habitats frequented by pollack for extended periods. This indicates that when pollack visited coastal receivers, they showed a propensity to remain in coastal habitats for extended periods, emphasizing the ecological significance of these areas for the species.

#### 3.2.3 Spatial network graphs

**FIGURE 7:**
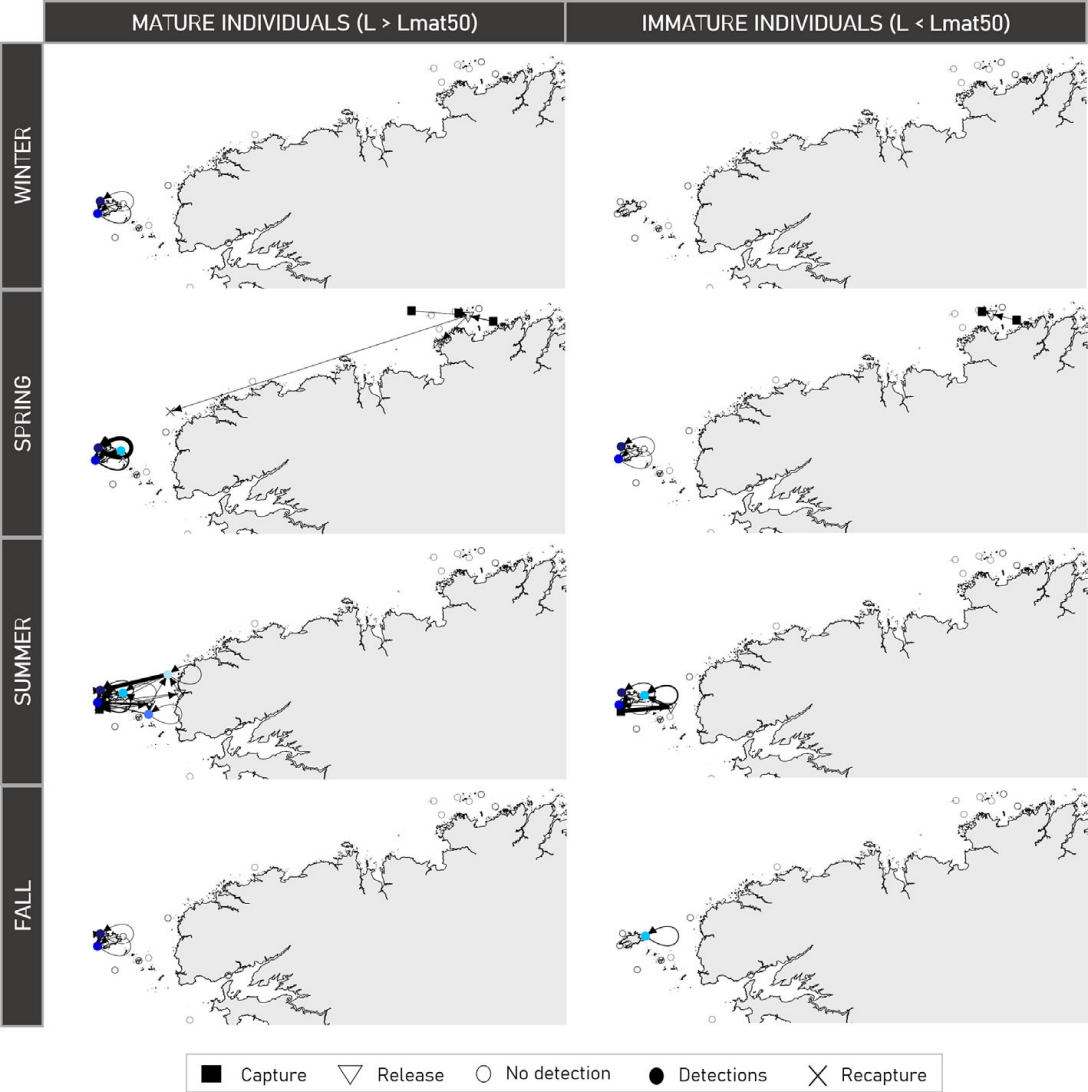
Seasonal movements of mature and immature fish inferred by capture, release, acoustic detections, and recapture locations. The direction of movements is indicated by the arrows. The width of arrows is proportional to the number of fish that moved between sites. Open circles are positions of ETN acoustic receivers where no detections occurred. Winter: December, January, February. Spring: March, April, May. Summer: June, July, August. Fall: September, October, November. The length at maturity *Lmat*_50_ = 43.7 cm was retrieved from Alemany (2017).

Seasonal movements of pollack inferred from the capture, release, and recapture positions, as well as acoustic detections, are contrasted between mature and immature individuals (Figure 7). Mature individuals displayed movements throughout the year and moved at larger scale than immature fish, especially during spring and summer. One mature individual traveled between the Côtes d'Armor and the Iroise Sea during spring (fish A19230/22060608). In addition, immature pollack were detected by single receivers, while mature pollack were frequently observed to move between receivers within the Iroise Sea. These results are in line with the contrasted residency patterns observed for mature and immature fish (Figure 6).

### 3.3 Movements inferred from acoustic and data storage tags

#### 3.3.1 Temperature and depth recorded

As of October 2023, 10 out of 48 double‐tagged pollack have been recaptured (21%), and all the archival tags have successfully recorded temperature and depth data. Notably, all the fish recaptured in the Iroise Sea were tagged in the Iroise Sea, except fish A19230/22060608, which was tagged in the Côtes d'Armor and subsequently recaptured in the Iroise Sea. Fish A19226/22060612 was tagged in the Côtes d'Armor, and the archival tag was found washed up on a beach nearby about a year later, suggesting that the individual died before the tag drifted ashore. The other nine DSTs were recovered by recreational or professional fishermen on capturing the fish. Six fish were recaptured during or at the end of the summer of 2022, while the remaining four were recaptured in the subsequent spring and summer (A18857/22060471, A18828/22060378, A19056/22060500, and A19226/22060612) (Table 3).

**TABLE 3:** Overview of the acoustic and archival data for the recaptured pollack.

Archival tag ID	Acoustic tag ID	Days detected	Archived days	Depth (m)	Temperature (°C)	Distance traveled (km)	Diffusion coefficient (σ)	Track sensitivity (km)
A19124	22060372	1	12 [13/06/22–24/06/22]	7.73 [1.37–36.62]	16.4 [5.91–22.8]	85.1 (80.3)	2.26 (2.38)	1.28 [0–7.71]
A19230	22060608	0	29 [19/05/22–16/06/22]	24.1 [2.43–105.9]	15.6 [9.61–31.0]	184	1.43	–
A18831	22060633	1	58 [17/06/22–13/08/22]	37.4 [0.81–92.93]	15.35 [4.01–27.1]	133 (115)	0.84 (0.65)	4.98 [0–22.8]
A18844	22060642	0	41 [17/06/22–27/07/22]	18.4 [0.75–83.1]	17.3 [12.8–27.0]	112	0.97	–
A18832	22060511	20	79 [17/06/22–03/09/22]	36.0 [0.37–99.4]	13.8 [12.8–29.7]	250 (263)	0.57 (1.13)	56.5 [0–91.8]
A19051	22060504	1	60 [13/06/22–11/08/22]	9.98 [1.68–33.06]	12.0 [2.08–30.14]	120 (122)	0.67 (0.67)	2.36 [0–11.1]
A18857	22060471	2	341 [16/06/22–22/05/23]	41.0 [2.06–106]	12.9 [10.6–16.1]	1055 (914)	1.36 (1.98)	12.6 [0–103]
A18828	22060378	10	372 [17/06/22–23/06/23]	23.7 [5.12–78.75]	13.1 [10.9–16.0]	1828 (1034)	1.66 (0.97)	19.2 [0–78]
A19056	22060500	4	435 [13/06/22–21/08/23]	56.8 [1.93–186]	13.0 [10.8–16.1]	1570 (2125)	1.14 (1.57)	23.5 [0–198]
A19226	22060612	0	447 [19/05/22–30/07/23]	47.6 [3.75–179]	13.6 [9.53–18.9]	1165	1.21	–

*Note*: Numbers represent the mean values, with minimum and maximum values enclosed in square brackets. The first and last dates correspond to the release date and the presumed date of fish death, respectively. The distance traveled and the diffusion coefficient were estimated by the geolocation model fed with archival data and acoustic detections if they occurred. Values in brackets are outputs of the geolocation model without the integration of acoustic detections. Track sensitivity is computed as the distance between the tracks estimated with and without acoustic detections.

Seven out of the 10 recaptured fish were detected by the acoustic telemetry network. Among these, two (fish A19124/22060372 and A19051/22060504) were exclusively detected at the temporary stations Detection_6 and Detection_7 deployed in the Bay of Lampaul at Ouessant Island. All other detections of the recaptured fish occurred in the Iroise Sea, primarily near Ouessant Island during the summer season. Fish A18828/22060378 was the sole double‐tagged pollack to be detected by acoustic receivers outside the summer period, in November and April.

**FIGURE 8:**
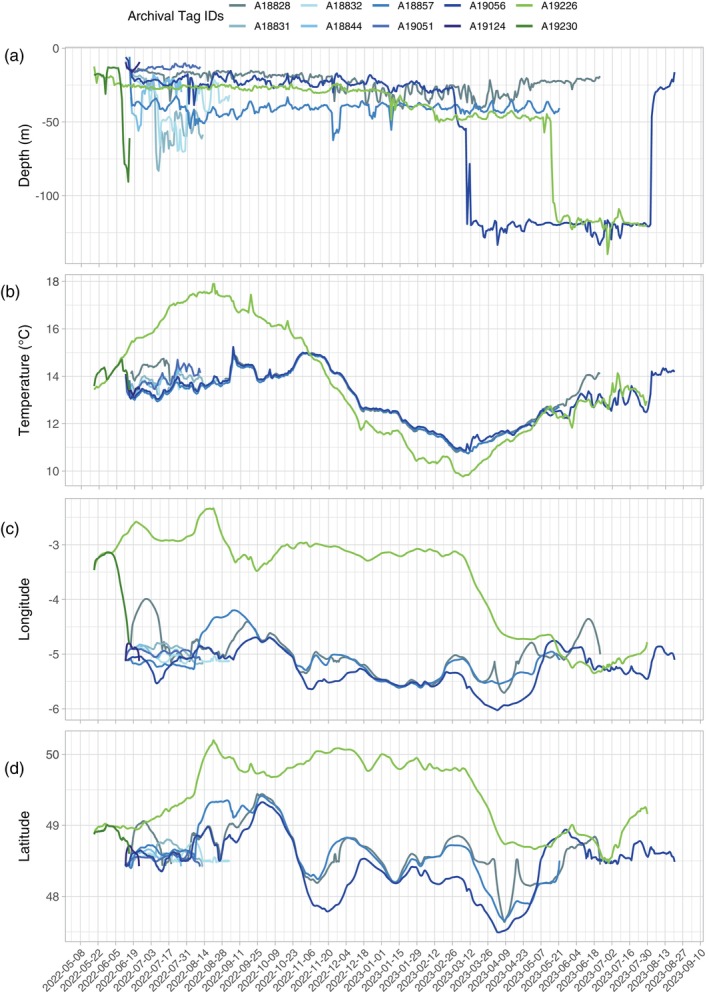
Mean daily depth (a) and temperature (b) data were recorded by the 10 recovered archival tags. Mean daily longitude (c) and latitude (d) were estimated using the geolocation model, which incorporates acoustic detections whenever they occurred.

Most of the time, fish were found at depths ranging from 0 to 80 m, except for two individuals (A19056/22060500 and A19226/22060612) observed at depths of up to 186 m during the spring and summer of 2023 (Figure 8a). These fish were observed between 20 and 30 m for a prolonged period, before transitioning to deeper waters close to 150 m depth on average for several months. Three individuals (fish A19124/22060372, A18844/22060642, and A19051/22060504) were found in shallow waters of <20 m depth on average for a period ranging from 12 to 60 days (Figure 8a and Table 3). This suggests that pollack could experiment with a wide gradient of bathymetric habitats over prolonged periods.

Temperature profiles were relatively consistent among the eight individuals tagged in the Iroise Sea, with temperatures ranging between 13 and 14°C in summer and between 11 and 13°C in winter (Figure 8b). The individual tagged and recaptured in the Côte d'Armor (fish A19226/22060612) exhibited higher temperature values during the summer of 2022, reaching temperatures of up to 18°C, and lower temperature values during winter, dropping down to 9.5°C. However, by the end of the time series in summer 2023, this fish displayed temperature profiles similar to the other fish. Notably, during June and July 2023, this individual showcased comparable depth and temperature profiles to fish A19056/22060500, suggesting shared habitat utilization during this period.

#### 3.3.2 Reconstructed tracks

**FIGURE 9:**
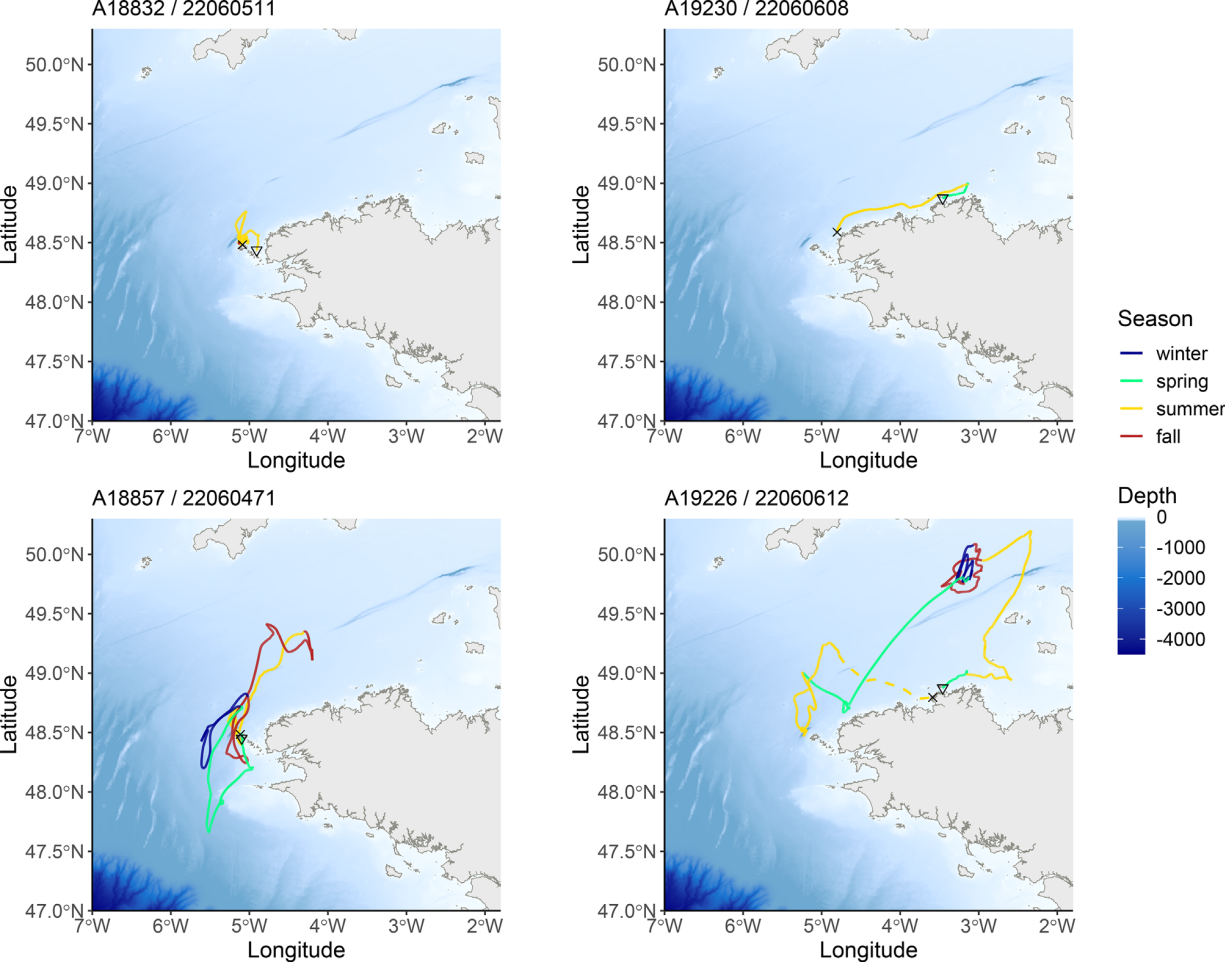
Track reconstructions of four recaptured fish. On the left, fish tagged, detected, and recaptured in the Iroise Sea. On the right, fish tagged in the Côtes d'Armor. These two fish were not detected by the telemetry network. Triangles and crosses denote release and recapture/recover positions, respectively. Dotted lines represent tag drifting after fish death at sea. Winter: December, January, February. Spring: March, April, May. Summer: June, July, August. Fall: September, October, November.

The longitudes and latitudes estimated by the geolocation model appeared quite consistent among the fish tagged in the Iroise Sea over the duration of the tracking period (Figure 8). Among the eight fish tagged in the Iroise Sea, all were consistently located within that area during the summer periods, suggesting a potential feeding area (Figure 9 and Appendix D in the Supporting Information). This observation aligns with the time series of acoustic detections (Figure 4).

Remarkably, fish A18857/22060471 and A18828/22060378 demonstrated similar movement patterns throughout the year (Figure 9 and Appendix D in the Supporting Information). Fish A19056/22060500 exhibited similar longitudinal and latitudinal patterns as the two later fish across the year. However, it was systematically found southwest compared to those fish (Figure 8). All three fish were predicted to have crossed the 48° parallel separating the Bay of Biscay from the Channel between mid‐March and late April. Fish A19056/22060500 also did an incursion into the Bay of Biscay during the fall of 2022. Finally, these three individuals showed incursions in the entrance of the western Channel during the fall.

For the two fish tagged off the Côtes d'Armor, directed movements towards the Iroise Sea were observed during the summers of 2022 (A19230/22060608) and 2023 (A19226/22060612) (Figure 9). After tagging in the Côtes d'Armor, the latter fish spent a significant amount of time in the central‐western Channel during the fall and winter before eventually moving into the Iroise Sea. These movements aligned with the incursions of fish A18857/22060471, A18828/22060378, and A19056/22060500 in the western Channel, but fish A19226/22060612 was found in the central part of the western Channel (Figure 9).

On average, pollack traveled 3.5 km/day (ranging between 2.0 km/day for fish A19051/22060504 and 7.1 km/day for fish A19124/22060372) (Table 3). No relationship was found between the distance traveled per day and the fish size (*R* ^2^ = −0.0713, *F* = 0.401, *p* = 0.5442).

#### 3.3.3 Validation of the geolocation model

The positional accuracy was assessed for the seven recaptured fish that were detected at least once by acoustic receivers, including those at temporary stations (Table 3). On average, the discrepancy between positions estimated by the geolocation model with and without acoustic ranged between 1.28 and 56.5 km. However, fish A19056/22060500 notably showed a positional accuracy discrepancy of up to 198 km. As a general trend, we observed that fish detected more frequently displayed a higher maximum distance between the estimated tracks with and without acoustic information.

## 4 DISCUSSION

Understanding the spatial ecology of fish populations is crucial for effective management and conservation strategies. The present work was the first investigation of the spatial dynamics of the pollack population in the northeast Atlantic, employing a combination of two innovative tracking technologies: acoustic telemetry and archival tagging. Despite the challenges posed by the sensitivity of pollack to depressurization, our study successfully implemented double‐tagging methods, allowing us to track pollack within the Channel. This research revealed initial evidence of seasonal movements and habitat use among pollack and shed light on size‐specific movement behaviors. Overall, >40% of the fish were detected by the telemetry network and 21% of the double‐tagged fish were recaptured a year after being released. This demonstrates the relevance of such a combination of tracking methods for monitoring the movements of the under‐studied pollack.

### 4.1 Feasibility of double‐tagging for pollack

Sudden depressurization may decrease fish survival if they are unable to regulate their swim bladder's air pressure after being caught at depth and rapidly brought to the surface (Muoneke & Childress, 1994). Pollack, like other Gadidae species (Ferter et al., 2015; Nichol & Chilton, 2006), is known to be sensitive to barotrauma during capture, which may limit the post‐tagging survival. In this study, we tested two different strategies for tagging pollack in France and the UK. In France, pollack were captured early in the morning at a maximum depth of 15 m and individuals were kept on board for several hours between being caught and being released at sea. Only individuals in healthy condition were selected (<10 fish were discarded). In the UK, fish were captured deeper, at an average depth of 25 m. Because difficulties in fish survival were encountered in the UK, the strategy was to minimize the time spent at the surface. Consequently, pollack were kept on board and tagged in <10 min. These differences in tagging protocols may explain the difference found in survival rates, and consequently in detection rates, between fish tagged in France and the UK. In France, pollack showed a remarkable long‐term survival rate after tagging, with 32 (46%) fish detected by the telemetry network at least once or recaptured. The short‐term post‐tagging experiment, conducted on a subset of 35 pollack in the Bay of Lampaul at Ouessant Island, showcased exceptionally high short‐term survival rates, reaching 97% within a few days after release and 54% on the long‐term. Our observations did not reveal any difference in post‐tagging survival between fish tagged with one or two tags, nor was any correlation found with fish size. In Cornwall, 23% of the pollack were detected after release, which may suggest that fish left the area or did not survived the tagging procedure.

Recently, a tagging experiment on pollack using acoustic transmitters inserted in the abdominal cavity was carried out along the Norwegian coast in shallow waters (Freitas et al., 2021), following a tagging protocol initially developed for cod (Olsen et al., 2012). No mortality was observed, whatever the size of the individuals considered (35–52 cm). In another tagging experiment along the Spanish coast, Mucientes et al. (2021) tagged a single pollack, likely a juvenile, captured in shallow waters (<10 m) using a hand net during scuba diving. The acoustic transmitter was successfully inserted into the abdominal cavity. The individual survived the experiment and was recaptured 10 km south of the study area after a year at sea.

Collectively, these findings suggest that the optimal practice for tagging pollack involves capturing them at shallow depths to minimize rapid depressurization and observing a recuperation period in a tank after tagging.

### 4.2 Benefits from combining acoustic telemetry with archival tagging

The present study unraveled the movement trajectories of 10 pollack, among which seven fish were detected by the acoustic telemetry network. Classical DST technologies require that the tag is recovered a posteriori, which obviously limits the quantity of sensor data that can be retrieved and ultimately the number of fish that can be tracked. Conversely, archival tags provide valuable insights into the movements of marine animals beyond the coverage of the coastal acoustic telemetry network. Consequently, integrating acoustic detections in the geolocation model was an opportunity to compensate insufficient resolution of environmental fields in coastal areas where deploying acoustic receivers was convenient (Goossens et al., 2023; Liu et al., 2017).

On average, the positions estimated by the geolocation model with and without acoustic detections exhibited relatively similar results, with an average track sensitivity of 17.2 km among the seven detected fish, ranging from 0 km up to 198 km for fish A19056/22060500 (Table 3). These results are of the same order of magnitude as the track sensitivity estimated by Goossens et al. (2023) for European seabass, Atlantic cod and starry smooth‐hound.

**FIGURE 10:**
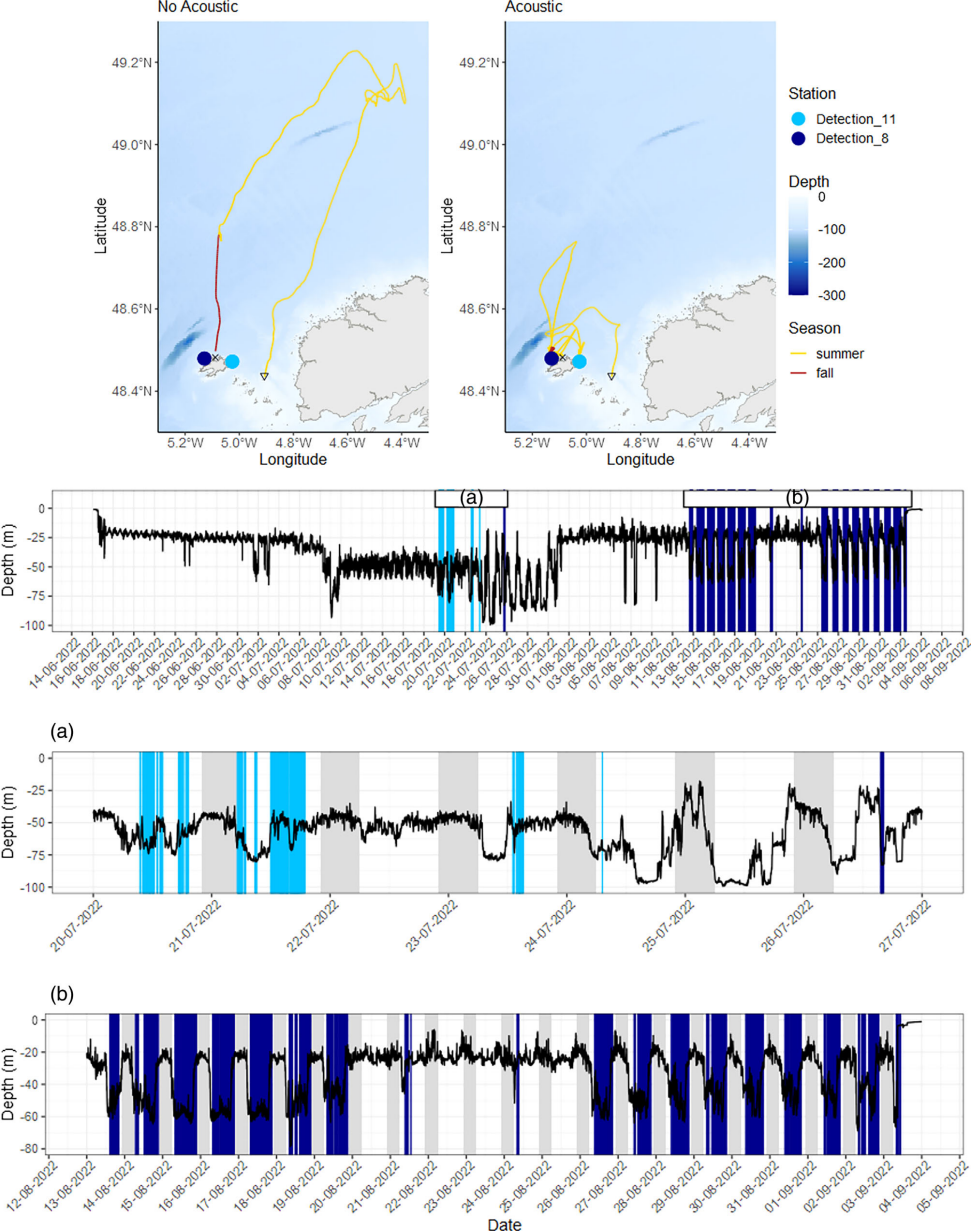
Track reconstructions of fish A18832/22060511 with or without acoustic detections and the depth time series recorder by the DST. (a) and (b) Specific periods when acoustic detections occurred at station Detection_11 and Detection_8 near Ouessant Island. Vertical lines indicate acoustic detections. Gray rectangles are night time. Triangles and crosses denote release and recapture positions, respectively.

In general, the distances traveled were comparable between the two types of reconstructed tracks (with and without acoustic). However, fish A18828/22060378 was estimated to have traveled approximately 800 km more when the acoustic detections were included in the model. Conversely, fish A19056/22060500 was estimated to have traveled about 500 km less when the acoustic detections were integrated. Differences observed between the tracks, whether including or excluding acoustic detections, could be explained by the fact that acoustic detections sometimes occur after a prolonged period with no detections. This may force the trajectory to shift back towards the coast while previously estimated offshore. For example, fish A18832/22060511 exhibited the highest average track sensitivity (56.5 km) among the recaptured fish, possibly due to its significant number of detections (>1900 detections over 20 days) (Table 3 and Appendix C in the Supporting Information). The fish was regularly detected at station Detection_11 approximately a month after its release (Figure 10). The fish was then detected at station Detection_8 regularly during daytime for a week between August 13 and 20, and again between August 26 and September 3, before being recaptured near the receiver. The track of fish A18832/22060511 was sensitive to the integration of acoustic detections (Figure 10). When the track was inferred solely from the archival tag data and the release/recapture positions, the fish appeared to have moved northward, towards the entrance of the western Channel, before returning to Ouessant Island, where it was recaptured. Conversely, when the acoustic detections were added to constrain the model, the resulting path showed a more coastal trajectory in the vicinity of Ouessant Island. Notably, this fish sometimes exhibited a rhythmic diving behavior, diving during the day (as indicated by detections from the receiver placed at around 60 m depth) and ascending at night. Dedicated analyses of pollack diving activity are required to unravel the drivers of such dial diving patterns (Heerah et al., 2017).

Solely relying on archival information might have overlooked coastal movements, potentially keeping the fish offshore if the depth and temperature gradients recorded were insufficient to accurately differentiate between offshore and coastal positions. This limitation is closely linked to the relatively coarse resolution of the reference field used in our geolocation model. Specifically, we used the Atlantic—European North West Shelf—Ocean Physics Analysis and Forecast, a hydrodynamic model providing sea surface height, sea water temperature, and bathymetry with resolutions of approximately 2 km (CMEMS, 2023). Although this study did not aim to test the sensitivity of estimated positions to the reference fields used, we anticipate that enhancing the resolution of reference fields and reducing positioning errors will significantly benefit from assimilating field observation data, such as in situ data collected by acoustic receivers (e.g., temperature measurement).

The main conclusion is that reference fields, which are model outputs, contain biases and errors that vary over time and space. These uncertainties persist when estimating fish positions using geolocation models. Acoustic data, similarly to recapture positions (Gatti et al., 2021), provide a unique opportunity to assess these uncertainties. Although incorporating these observations may distort the trajectories, it represents the best estimate available, therefore we recommend using the corrected trajectories. Note that the general patterns of trajectories, with or without acoustic data, remain largely unchanged, suggesting that ecological knowledge can still be drawn without knowing the exact fish location. The level of accuracy required depends on the scale of the ecological question. For instance, characterizing annual migration patterns may require less precision in trajectories compared to the analysis of daily habitat use on a feeding ground.

### 4.3 First insights into seasonal movements of pollack

#### 4.3.1 Movement extent

Pollack were mostly detected within a limited range of about 30 km from their release sites, both in Cornwall and the Iroise Sea. Surprisingly, no detections were recorded from the fish tagged in the Côtes d'Armor and Bay of Seine. This absence of detections could suggest that these individuals either moved to locations beyond the detection range of the network or experienced mortality during the monitoring period. These findings, solely relying on acoustic telemetry, highlight limited movements of pollack, consistent with previous pollack tracking studies. In Jakobsen (1985), the author applied external tags on 3–4‐year‐old pollack (28–52 cm) off the Norwegian west coast, and 23% of these fish were recaptured after 5 years, within a range of up to 20 miles (30 km) from the release location, suggesting a lack of evidence for long‐range movement. A decade later, Sarno et al. (1994) equipped juvenile pollack (24–36 cm) with acoustic transmitters placed in the stomach in a Scottish sea loch, revealing that young pollack were solitary, slow‐moving, and exhibited restricted movements within the sea loch. Similarly, Mucientes et al. (2021) recaptured a juvenile pollack approximately 10 km away from the release site about a year after tagging. Even if those studies partly focused on immature pollack, they tended to indicate that pollack do not exhibit large‐scale movements.

Archival tags bring complementary information on pollack movements beyond the coastal telemetry network. From the 10 recaptured tags, the geolocation model effectively did not estimated movements between the coasts of Brittany in France and Cornwall in the UK. As previously mentioned, no fish tagged in the Côtes d'Armor was detected, but two of those individuals appeared to have moved based on the estimated tracks from the geolocation model: fish A19230/22060608 was recaptured north of the Iroise Sea, approximately 100 km away from the tagging location, shortly after tagging, while fish A19226/22060612 likely journeyed to the Iroise Sea during the summer of 2023 and died at sea, before the tag was retrieved in the Côtes d'Armor, where it was initially tagged (Figure 9). Overall, archival tags revealed that pollack mobility might be slightly higher than previously observed by acoustic telemetry or classical mark‐recapture studies. This contrasting result might be related to the fact that we reconstructed the trajectories of large pollack (total length ranging between 460 and 619 mm), whereas other tracking studies mostly focused on immature pollack (e.g., Sarno et al., 1994; Mucientes et al., 2021). By reconstructing pollack trajectories using a geolocation model, we found that individuals may have traveled up to 1800 km in about a year, with an average daily displacement of 3.5 km. In comparison, using similar geolocation modeling, de Pontual et al. (2019) estimated that European seabass (*Dicentrarchus labrax*) covered an average of 12.1 km/day (ranging between 5 and 29 km/day). Another Gadidae species, the Atlantic cod (*Gadus morhua*), showed a relatively similar daily speed as pollack, estimated at 4 km/day on average (Liu et al., 2017). Although these numbers depend on migratory behavior (e.g., migrant or resident) and fish size, our results suggest that pollack is a relatively slow‐moving bentho‐demersal fish species.

Regarding the absence of acoustic detections in Cornwall for fish tagged along the French coast, one cannot eliminate the possibility that a fish tagged with an open protocol (OPI) acoustic transmitter might have traveled through the Channel (which seems unlikely, given the reconstructed tracks), but remained undetected due to the differences in the acoustic protocols used on receivers in the UK (R64K). This discrepancy in acoustic protocols could hinder our capacity to track fish movements across different jurisdictions, potentially leading to a significant bias in our comprehension of fish population spatial dynamics. This emphasises the need for standardizing open protocols within the European Tracking Network (Reubens et al., 2021).

#### 4.3.2 Movement seasonality

Acoustic telemetry revealed seasonal patterns in detections, indicating a peak in activity during summer in the Iroise Sea. Confirmation from archival tags emphasized the significance of the Iroise Sea as a habitat of interest for pollack, particularly during summer. This was evident as all recaptured fish, regardless of whether they were tagged in the Iroise Sea or the Côtes d'Armor, were found to frequent this area during the summer months. This may suggest a potential role of the Iroise Sea as a feeding area for the species.

Four archival tags recorded approximately a year at sea (fish A19226/22060612, A18857/22060471, A18828/22060378, and A19056/22060500). Fish A18857/22060471, A18828/22060378, and A19056/22060500 exhibited notably similar trajectories (Figure 9 and Appendix D in the Supporting Information). All displayed a consistent pattern of remaining within or near the Iroise Sea throughout the year, with excursions into the western Channel entrance during the fall. Subsequently, they migrated southwest of Ouessant Island during the winter and spring months. This similarity in individual two‐dimensional trajectories hints at potential collective movement behaviors and/or shared environmental conditions driving their movements. While those fish shared similar conditions of temperatures throughout the time series, they experienced different depth profiles (Table 3 and Figure 8), suggesting that they were not following the exact same path on the vertical dimension. As more archival tags are retrieved, elucidating the abiotic and biotic drivers of pollack movements will help refine our understanding of collective movement behaviors and population connectivity (Lowerre‐Barbieri et al., 2021; Lubitz et al., 2022).

Fish A19226/22060612 was tagged in the Côte d'Armor and its archival tag was recovered on a beach close to the tagging site. The geolocation model revealed that this individual spent its first summer off the Côtes d'Armor near the Channel Islands, moving northward towards the middle of the western Channel during the fall and winter, before transitioning to the Iroise Sea during the spring and summer of 2023 (Figure 9). While visiting the Iroise Sea during the summer of 2023, this fish was found up to 179 m, presumably in an underwater canyon northwest of Ouessant Island, before it died at the end of July 2023. The tag then drifted towards the beach, where it was found. Interestingly, it was not the only pollack that visited the deep underwater canyon near Ouessant Island at this period since fish A19056/22060500 showed a similar diving profile (Figure 8), and was estimated to have visited the same area using the geolocation model (Figure 9). These fish visited a diverse range of bathymetric habitats over prolonged periods of several months (Figure 8), indicating that pollack may shift from shallow to deep habitats.

Although describing seasonal movement patterns based on only four 1‐year tracks is challenging, these patterns appeared consistent with known pollack distribution from landings. According to Foucher (2017), the majority of landings in the ICES divisions 6 (Celtic Sea) and 7 (English Channel) occur typically between January and May, peaking in March, likely indicating the aggregation of individuals on spawning grounds. Analyzing 2013 landings from the southern Bay of Biscay to the southern English and Irish coasts, Leaute et al. (2017) identified a distribution from the middle of the Bay of Biscay to Cornwall, with a peak from southern Brittany to the entrance of the western Channel. This distribution, inferred from landings, perfectly aligns with the reconstructed tracks of the four pollack that spent a year at sea. Fish A19226/22060612 exhibited a migration pattern in the entrance of the western Channel during winter, potentially corresponding to the hypothesized breeding area suggested by Leaute et al. (2017). These authors also indicated the existence of another potential spawning area situated south of Brittany, which may correspond to the southern movements of fish A18857/22060471, A18828/22060378, and A19056/22060500 between mid‐March and late April. These pollack were estimated to cross the 48° parallel, moving from the Channel to the Bay of Biscay during a period that may overlap with the known reproduction period (Moreau, 1964).

At this stage of the research, it is premature to delineate the exact spawning grounds of pollack in the Channel. Nevertheless, observations indicate the potential significance of the Iroise Sea as a feeding area during the summer, akin to other fish species, such as seabass (de Pontual et al., 2019). Additional tagging surveys in the future will certainly add to our understanding of pollack's seasonal movements and essential habitats.

#### 4.3.3 Size‐specific movements

Based on acoustic detections, distinct movement patterns emerged between mature and immature pollack. In contrast to mature individuals, immature fish exhibited no detections between December and April, nor in August and October (Figure 5). Furthermore, immature pollack displayed fewer and shorter residency periods compared to larger individuals (Figure 6). This suggests that when immature fish were present within the network, they tended to remain in close proximity to the receivers over a short period before they eventually moved beyond the coverage of acoustic receivers. In contrast, mature fish demonstrated a higher number of residency periods for longer duration, indicative of frequent back‐and‐forth movements in the vicinity of the receivers (Figure 6). Lastly, irrespective of the season, immature pollack did not move between receivers and were exclusively detected by single receivers (Figure 7). Taken together, these findings may indicate that immature fish exhibit lower mobility than mature fish and tend to occupy a more restricted habitat. These observations align with the outcomes of previous studies on juvenile pollack tagging (Mucientes et al., 2021; Sarno et al., 1994). Another hypothesis explaining the different residency patterns observed could be that immature and mature pollack may partially inhabit different habitats. Our acoustic telemetry network has been designed in collaboration with fishermen. Since fishermen preferentially exploit larger fish, this may suggest that acoustic receivers were placed in habitats more favorable for mature fish than for immature ones, explaining the more frequent and longer residency periods of mature fish.

Size‐specific movement patterns are common among marine fish as different life stages occupy varying habitats throughout their life cycle (Carruthers et al., 2015; Goossens et al., 2020; Lowerre‐Barbieri et al., 2016, 2021). While immature individuals are frequently observed in nearshore nurseries where the predation risk is reduced (Dahlgren & Eggleston, 2000), larger fish often explore larger areas for feeding and spawning (Pittman & McAlpine, 2003). According to Moreau (1964) and Alonso‐Fernández et al. (2014), larger pollack are found more offshore than juveniles, which could be related to ontogenetic shift in fish distribution. The size‐specific behavior observed in pollack is also consistent with other Gadidae species, such as the Atlantic cod (Olsen et al., 2023). Indeed, Olsen et al. (2023) found a positive correlation between female body size and a connectivity index, suggesting that larger females are more mobile than smaller ones. Such size‐specific movement behavior could translate into management measures to protect larger individuals, ultimately improving the resilience of fish populations as large individuals utilize a diversity of habitats.

Since we did not double‐tag immature pollack to respect the 2% of tag‐to‐body‐mass ratio, we did not elucidate the movements of immature individuals during winter when they were absent from the telemetry network. Given the advances in tag miniaturization (Hussey et al., 2015), there is potential in the future to double‐tag immature fish, enabling a deeper understanding of size‐specific movement patterns and improved management of the pollack population.

### 4.4 Implication for management

This study is the first investigation of the spatial dynamics of pollack within the Channel area. Recently, Foucher (2017) highlighted the necessity of a tagging study to comprehend pollack movement patterns in the northeast Atlantic to ascertain potential movements between the Channel and the Bay of Biscay. Our findings revealed pollack movements within the Western Channel and highlighted the significance of the Iroise Sea as a feeding area during summer. Also, we estimated movements between the Channel (ICES division 7) and the Bay of Biscay (ICES division 8) for three individuals in spring 2023. However, it is premature to consider those movements as oriented spawning migrations based on solely three tracks. Low but significant genetic differentiation of pollack between the Bay of Biscay and the Channel was previously highlighted (Charrier et al., 2006) and aligned with the independent landing trends observed in the Bay of Biscay and the Channel (ICES, 2023a, 2023b). These findings would suggest independent dynamics in the Channel and Bay of Biscay, in contradiction with the southward movements estimated in our study. Nevertheless, our study was preliminary, and further insights into pollack spatial dynamics will emerge with ongoing data collection efforts. Conducting tagging experiments focusing on the south of Brittany in the Bay of Biscay and the southwest of the UK and Irish coasts would offer valuable complementary insights into pollack movements. This effort will aid in resolving the potential mixing between pollack from the Channel and the adjacent Bay of Biscay and Celtic Sea stocks.

Given the current gap of information about the spatial population dynamic of pollack in the Channel, recent ICES scientific advice recommended zero catch, both for professional and recreational fishing, owing to the concerning decline in landings in recent years (ICES, 2023b). Such an abrupt recommendation emphasizes the urgent need for expanded research programs on under‐studied species like pollack. Tracking free‐ranging fish, particularly those receiving minimal attention, is a pertinent approach to inform conservation policies and management decisions (Hays et al., 2019).

## 5 CONCLUSION

This study demonstrated the feasibility of double tagging for pollack and the high residency of individuals in coastal areas for feeding during summer. Movement patterns associated with spawning migrations were not elucidated at this stage and would require multi‐year acoustic detections and the recovery of additional archival tags. We stress that further acquisition of tracking data will improve the ecological understanding of pollack spatial population dynamics and refine current assessment model and local management strategies for this under‐studied, though harvested, species.

## AUTHOR CONTRIBUTIONS

M.G., M.W., and M.L. designed the methodology. M.G., J.M., J.M.D., and T.O. ran the analyses. M.W., M.L., S.M., F.G., C.L., M.D., T.S., P.D., and A.H. participated in the tagging experiments. M.W. and E.S. raised funding. M.G. wrote the original manuscript. All authors contributed to revising and editing the manuscript.

## Supporting information


**Appendix A** Supporting Information.

**Appendix B** Supporting Information.

**Appendix C** Supporting Information.

**Appendix D** Supporting Information.

## Footnotes

^1^
Note that total fish length was not available for fish tagged in Cornwall, so that the three fish detected in Cornwall were excluded from the subsequent analysis.
