# Supplementary material for: Combining acoustic telemetry with archival tagging to investigate the spatial dynamic of the understudied pollack, Pollachius pollachius
Source: J Fish Biol. 2024 Apr 25;106(5):1400–21. doi: 10.1111/jfb.15750 (PMC12120336; doi:10.1111/jfb.15750)
Supplement: Supplementary file 1 — Appendix A Supporting Information. [file JFB-106-1400-s001.pdf]

Journal of Fish Biology - Appendix A

Combining acoustic telemetry with archival tagging to investigate the spatial dynamic of the understudied pollack, *Pollachius pollachius*

Marine Gonse, Martial Laurans, Justus Magin, Tina Odaka, Jean-Marc Delouis, Stéphane Martin, François Garren, Coline Lazard, Mickael Drogou, Thomas Stamp, Peter Davies, Alice Hall, Emma Sheehan, and Mathieu Woillez

1 Quality Control procedure applied to acoustic detections

False detections resulting from signal collision or background noise can lead to erroneous indications of animal presence. We developed a filtering procedure to remove such false detections by adapting a method described by Hoenner et al. (2018). We established a set of quality control (QC) criteria. Each detection was scored based on the following criteria:

- **Isolated detections** - A detection failed this test if an acoustic tag was detected only once within a 5-hour time bin. Such isolated detections were deemed unrealistic, particularly for mobile species with a moderate movement speed, such as pollack.
- **Velocity** - A detection failed this test if the movement speed between two consecutive detections exceeded a species-specific threshold. For pollack, the velocity threshold was set at 3 meters per second.
- **Distance from release** - A detection failed this test if the distance between the acoustic receiver and the tagging release location exceeded a species-specific threshold. For pollack, this threshold was set at 1000 km.
- **Release date** - A detection failed this test if it occurred prior to the animal's tagging release date.
- **Distance** - A detection failed this test if the distance between two consecutive detections exceeded a threshold corresponding to the size of the network, which, in this case, was 700 km.
- **Signal to noise ratio** - A detection failed the test if the signal to noise ratio fell below a threshold. This information was provided by Thelma Biotel acoustic receivers. The threshold has been fixed to 10, meaning that the acoustic signal from the tag (the acoustic tag ID and timestamp) must represent at least 10% of the acoustic signal received by a receiver, with the rest being attributed to the ambient noise.

For all the criteria outlined above, if a detection failed the test, the criterion value was assigned to 2. If a detection passed the test, the criterion value was set to 1. If no information was available, the criterion value was set to 3. Subsequently, a final detection QC was computed to determine whether a given detection should be considered a true or false detection. A detection was valid ($QC = 1$) if the six tests were passed. A detection was considered likely valid ($QC = 2$) if five tests were passed. A detection was considered as likely invalid ($QC = 3$) if four tests were passed. A detection was considered invalid ($QC = 4$) if three or less tests were passed. As proposed by Hoenner et al. (2018), likely invalid and invalid detections were removed from the database.

References

Hoenner, X., C. Huveneers, A. Steckenreuter, C. Simpfendorfer, K. Tattersall, F. Jaine, N. Atkins, R. Babcock, S. Brodie, J. Burgess, et al. (2018). Australia's continental-scale acoustic tracking database and its automated quality control process. *Scientific data* 5(1), 1–10
