# Supplementary material for: Combining acoustic telemetry with archival tagging to investigate the spatial dynamic of the understudied pollack, Pollachius pollachius
Source: J Fish Biol. 2024 Apr 25;106(5):1400–21. doi: 10.1111/jfb.15750 (PMC12120336; doi:10.1111/jfb.15750)
Supplement: Supplementary file 2 — Appendix B Supporting Information. [file JFB-106-1400-s002.pdf]

Journal of Fish Biology - Appendix B

Combining acoustic telemetry with archival tagging to investigate the spatial dynamic of the understudied pollack, *Pollachius pollachius*

Marine Gonse, Martial Laurans, Justus Magin, Tina Odaka, Jean-Marc Delouis, Stéphane Martin, François Garren, Coline Lazard, Mickael Drogou, Thomas Stamp, Peter Davies, Alice Hall, Emma Sheehan, and Mathieu Woillez

1 Sensitivity analysis of the threshold of residency duration

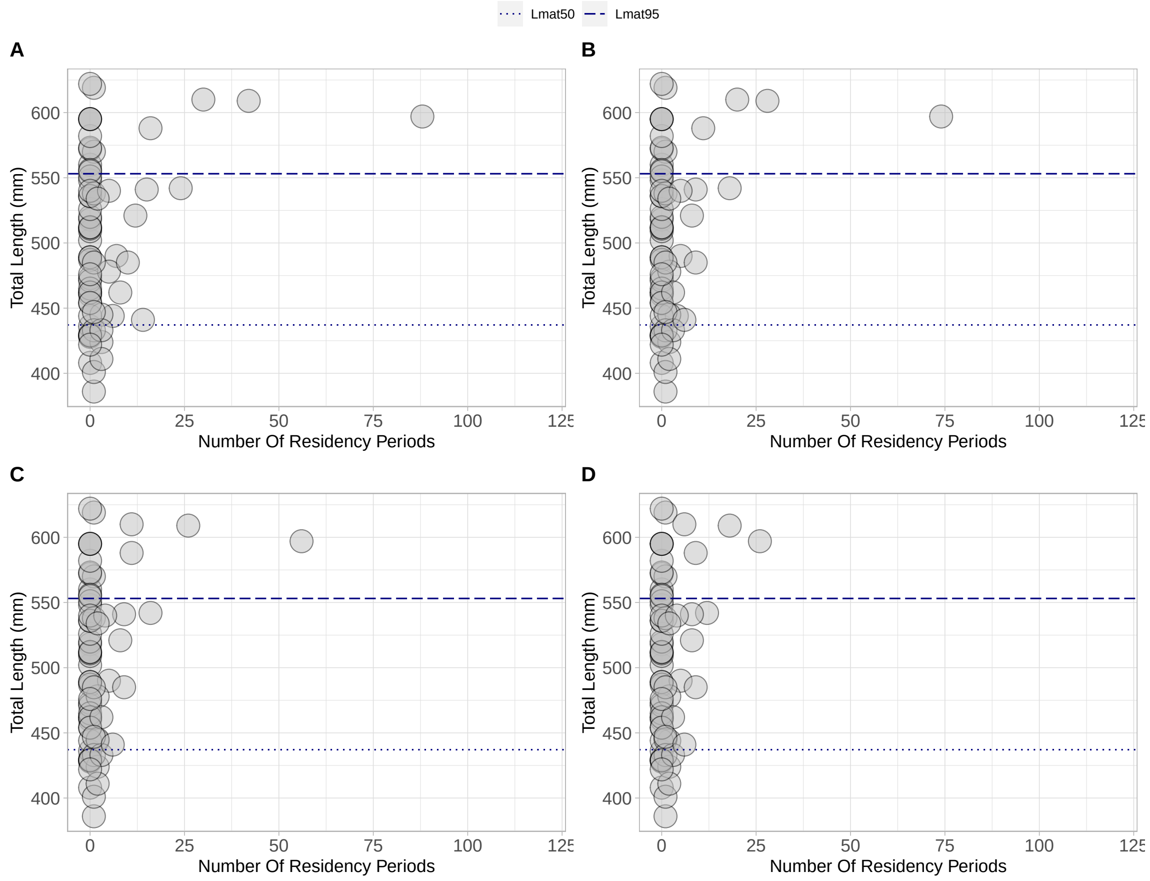

Figure 1: Relation between total fish length and number of residency periods with varying thresholds for residency duration: (A) 1-hr, (B) 6-hrs, (C) 12-hrs, and (D) 24-hrs.
