# Supplementary material for: Combining acoustic telemetry with archival tagging to investigate the spatial dynamic of the understudied pollack, Pollachius pollachius
Source: J Fish Biol. 2024 Apr 25;106(5):1400–21. doi: 10.1111/jfb.15750 (PMC12120336; doi:10.1111/jfb.15750)
Supplement: Supplementary file 3 — Appendix C Supporting Information. [file JFB-106-1400-s003.pdf]

Journal of Fish Biology - Appendix C

Combining acoustic telemetry with archival tagging to investigate the spatial dynamic of the understudied pollack, *Pollachius pollachius*

Marine Gonse, Martial Laurans, Justus Magin, Tina Odaka, Jean-Marc Delouis, Stéphane Martin, François Garren, Coline Lazard, Mickael Drogou, Thomas Stamp, Peter Davies, Alice Hall, Emma Sheehan, and Mathieu Woillez

1 Number of detections per pollack, *Pollachius pollachius*

A total of 83 pollack have been tagged with acoustic transmitters in 2022, as part of the Fish Intel project. A total of 19719 detections have been recorded as of December 2023. Hereafter we present the detail of the number of detections per fish.

Table 1: Number of detections per pollack

Acoustic tag IDs	Archival tag IDs	Number of detections
22043765	NA	0
22043766	NA	0
22043767	NA	230
22043768	NA	0
22043769	NA	0
22043770	NA	0
22043771	NA	0
22043772	NA	0
22043773	NA	5
22043775	NA	0
22060636	A18931	0
22060646	A18925	0
22060657	A18926	0
22060608	A19230	0
22060612	A19226	0
22060613	A19234	0
22060616	NA	0
22060647	A19237	0
22060649	NA	0
22060651	NA	0
22060582	A18892	0
22060584	A18895	0
22060586	A19217	0
22060590	A18884	0
22060476	NA	0
22060488	A18876	6
22060327	A19053	25
22060328	A19043	42
22060329	A19052	398
22060331	A19045	15
22060332	NA	1730
22060335	A19044	61
22060336	NA	164
22060362	NA	11
22060365	NA	9
22060370	NA	10
22060372	A19124	48
22060489	NA	10
22060500	A19056	219

22060504	A19051	2
22060654	A19050	11
22060431	A18872	9
22060472	A19041	5
22060435	A18859	11
22060445	NA	7
22060426	A18845	110
22060427	A19038	5
22060430	A18849	230
22060439	A18868	39
22060441	NA	22
22060444	A18854	175
22060451	NA	20
22060452	A18843	135
22060465	NA	52
22060471	A18857	10
22060449	NA	6
22060467	NA	6
22060468	NA	4
22060643	A18827	13
22060376	A18856	0
22060378	A18828	62
22060381	A18833	0
22060385	A18825	4
22060436	A18847	3604
22060438	NA	623
22060456	A18846	0
22060458	NA	102
22060459	A18855	0
22060469	A18837	22
22060511	A18832	1903
22060632	NA	0
22060633	A18831	3
22060634	NA	24
22060642	A18844	0
22060386	A19033	0
22060387	A19131	73
22060390	NA	2
22060393	A18848	0
22060406	NA	723
22060466	A18839	0
22114110	NA	2
22114105	NA	0
22114104	NA	0
