# Supplementary material for: Combining acoustic telemetry with archival tagging to investigate the spatial dynamic of the understudied pollack, Pollachius pollachius
Source: J Fish Biol. 2024 Apr 25;106(5):1400–21. doi: 10.1111/jfb.15750 (PMC12120336; doi:10.1111/jfb.15750)
Supplement: Supplementary file 4 — Appendix D Supporting Information. [file JFB-106-1400-s004.pdf]

Journal of Fish Biology - Appendix D

Combining acoustic telemetry with archival tagging to investigate the spatial dynamic of the understudied pollack, *Pollachius pollachius*

Marine Gonse, Martial Laurans, Justus Magin, Tina Odaka, Jean-Marc Delouis, Stéphane Martin, François Garren, Coline Lazard, Mickael Drogou, Thomas Stamp, Peter Davies, Alice Hall, Emma Sheehan, and Mathieu Woillez

1 Track reconstructions of six remaining recaptured fish

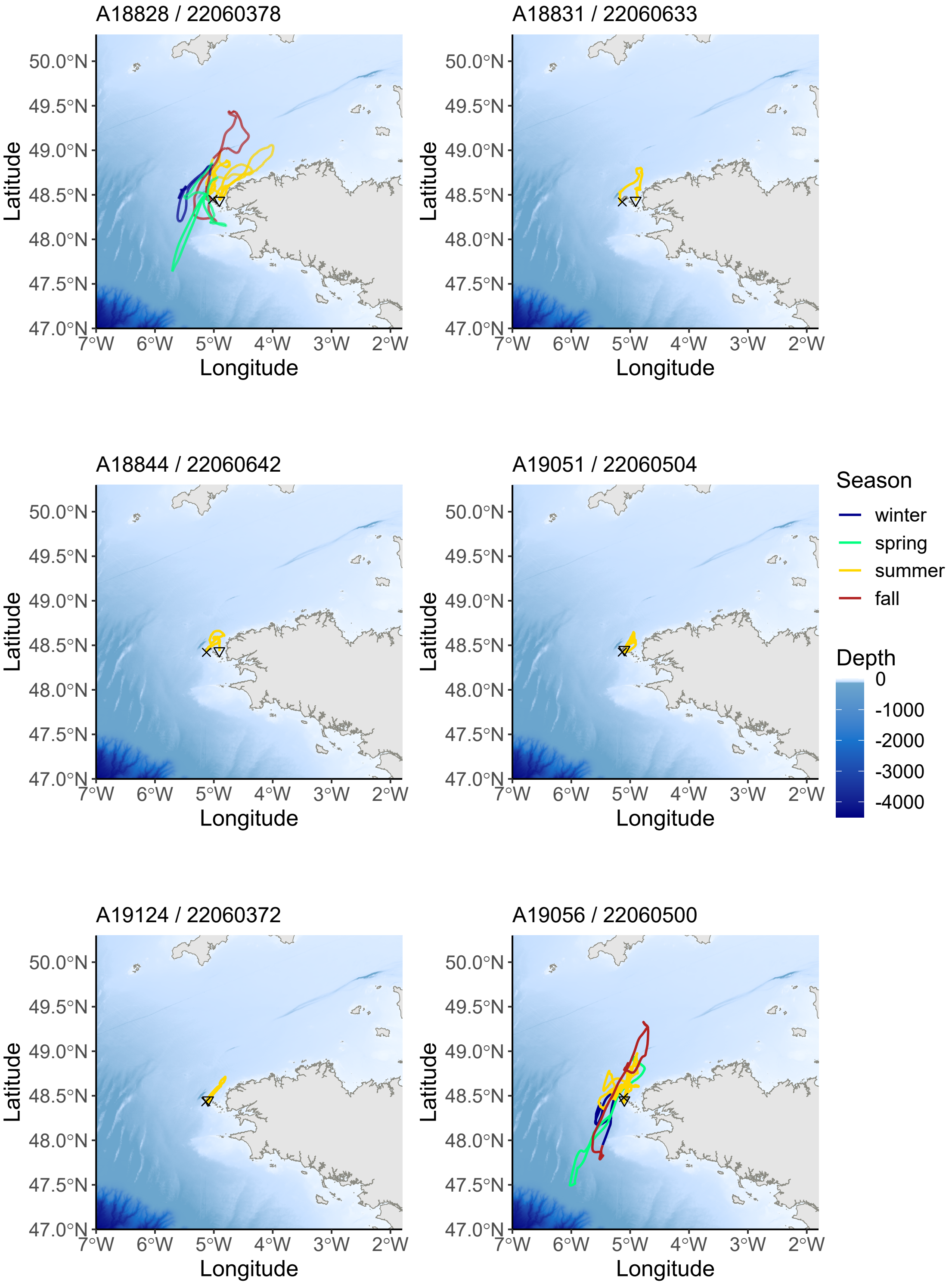

Figure 1: Track reconstructions of six recaptured fish that were previously tagged in the Iroise Sea. Triangles and crosses indicate release and recapture positions respectively.
